# *Pseudomonas* PA14H7: Identification and Quantification of the 7-Hydroxytropolone Iron Complex as an Active Metabolite against *Dickeya*, the Causal Agent of Blackleg on the Potato Plant

**DOI:** 10.3390/molecules28176207

**Published:** 2023-08-23

**Authors:** Euphrasie Munier-Lépinay, David Mathiron, Anthony Quéro, Mounia Khelifa, Sylvain Laclef, Serge Pilard

**Affiliations:** 1 inov3PT—Recherche Développement Innovation des Producteurs de Plants de Pomme de Terre, 43-45 Rue de Naples, 75008 Paris, France; euphrasie.lepinay@inov3pt.fr (E.M.-L.); mounia.khelifa@inov3pt.fr (M.K.); 2 Plateforme-Analytique (PFA), Institut de Chimie de Picardie FR 3085, Université de Picardie Jules Verne, 33 Rue Saint Leu, 80039 Amiens, France; david.mathiron@u-picardie.fr; 3 Laboratoire de Glycochimie, des Antimicrobiens et des Agroressources (LG2A), Institut de Chimie de Picardie FR 3085, Université de Picardie Jules Verne, 33 Rue Saint Leu, 80039 Amiens, France; 4 UMRT INRAE 1158 BioEcoAgro, UFR de Pharmacie, Université de Picardie Jules Verne, 1 Rue des Louvels, 80037 Amiens, France; anthony.quero@u-picardie.fr

**Keywords:** 7-hydroxytropolone, iron-complex, *Pseudomonas*, *Dickeya*, *Pectobacteriaceae*, biocontrol, blackleg, soft rot, potato

## Abstract

Soft rot *Pectobacteriaceae* (SRP), such as *Pectobacterium* and *Dickeya*, are phytopathogenic agents responsible for blackleg disease on several crops, such as potatoes, affecting the yield and depressing the seed production quality. However, neither conventional nor biocontrol products are available on the market to control this disease. In this study *Pseudomonas* PA14H7, a bacteria isolated from potato rhizosphere, was selected as a potential antagonist agent against *Dickeya solani*. In order to understand the mechanism involved in this antagonism, we managed to identify the main active molecule(s) produced by PA14H7. Cell-free supernatant (CFS) of PA14H7 cultures were extracted and analyzed using LC-MS, GC-MS, and NMR. We further correlated the biological activity against *Dickeya solani* of extracted CFS-PA14H7 to the presence of 7-hydroxytropolone (7-HT) complexed with iron. In a second time, we have synthesized this molecule and determined accurately using LC-UV, LC-MS, and GC-MS that, after 48 h incubation, PA14H7 released, in its CFS, around 9 mg/L of 7-HT. The biological activities of CFS-PA14H7 vs. synthetic 7-HT, at this concentration, were evaluated to have a similar bacteriostatic effect on the growth of *Dickeya solani*. Even if 7-HT is produced by other *Pseudomonas* species and is mostly known for its antibacterial and antifungal activities, this is the first description of its involvement as an effective molecule against pectinolytic bacteria. Our work opens the way for the comprehension of the mode of action of PA14H7 as a biocontrol agent against potato blackleg.

## 1. Introduction

Cultivated worldwide, the potato crop is sensitive to a large panel of pests and diseases. Among these, blackleg, also named soft rot when affecting tubers, is a bacterial disease caused by soft rot *Pectobacteriaceae* (SRP) from the genus *Pectobacterium* or *Dickeya*. SRP produce macerating enzymes that degrade the cell wall of the potato plant. Symptoms associated to blackleg are various, but the most common include yellowing and/or wilting of the aerial part and, typically, necrosis of the stem that can also affect tubers [1,2,3]. However, contrary to other diseases affecting the potato, no treatments are commercially available to control blackleg in vegetation and soft rot in conservation. Therefore, to guarantee the quality of seed potatoes, their production is monitored through strict specifications [4,5,6]. Consequently, blackleg is regularly the main cause underlying the downgrading or rejection of seed potato crops [7]. As a consequence, each year in France, 25% to 45% of rejection cases are due to blackleg, which contribute to an estimated average loss of 49 million euros for the European seed potato sector [8].

In order to control blackleg, different strategies are currently under investigation. One is based on the interaction between bacteria. Indeed, the production of macerating enzymes is led by virulence genes that are partially triggered through the quorum-sensing (QS) mechanism [9]. In other words, gene expression is coupled to bacterial cell concentration and is mediated by the diffusion of specific signal molecules. One of the best known QS-interrupting strategies is quorum quenching (QQ), which involves the enzymatic degradation of these signal molecules. The QQ strategy is effective for the *Pectobacterium* genus [10], but not for the *Dickeya* genus, as its virulence genes do not rely only on the QS mechanism [11]. Other studies using essential oils [12,13] or glycolipid-like compounds [14], only focused on controlling *Pectobacterium*, such as *P. atrosepticum*, and not on the genus *Dickeya*, which is known as the most damaging pathogen causing blackleg [15].

In this context, the challenge was to find a biocontrol solution against both genera responsible for the disease. Efforts were focused on the identification of bacteria which naturally colonized the same ecological niche as the pathogenic species [16]. Thus, six bacteria were isolated from the potato rhizosphere soil: *Pseudomonas fluorescens* PA3G8, *Pseudomonas brassicacearum* PA1G7 and PP1-210F, *Pseudomonas lactis* PA4C2, *Pseudomonas* sp. PA14H7, and *Bacillus simplex* BA2H3 [17]. Their antagonistic activities were first evaluated against *Pectobacterium atrosepticum* and *Dickeya dianthicola* [17,18]. As a second step, this evaluation was extended to a representative panel of *Pectobacterium* and *Dickeya* diversity in France, which showed that PA14H7, as well as PA1G7 and PP1-210F, had the greatest activity against SRP [19].

Nevertheless the mode of action against SRP [20] of the six antagonist strains cited above is not known. According to the literature, the *Pseudomonas* and *Bacillus* genera are able to produce a wide range of bioactive metabolites, such as lipopeptides, phenazines, phloroglucinols, pyrroles, tropolones, and volatile organic compounds, which are used as antimicrobial agents, siderophores, or biosurfactants [21,22,23,24]. In this study, we focused on the identification of secondary metabolites of interest in order to control blackleg disease caused by SRP.

For this purpose, we evaluated the activity of the six bacteria antagonists against SRP using the cell-free supernatants (CFS) rather than direct bacterial confrontation. Indeed, bacterial CFS has been reported to be potential source of biocontrol agents useful in sustainable agriculture [25]. The bacteria showing the most efficient activity was selected and its main active metabolite was characterized using the liquid chromatography coupled to mass spectrometry (LC-MS), gas chromatography coupled to mass spectrometry (GC-MS), and nuclear magnetic resonance (NMR) techniques. The metabolite was chemically synthesized in order to confirm its structure and biological activity and was used as a standard to quantify the amount produced by the bacteria in its CFS (ultra-violet (UV), LC-UV, LC-MS, and GC-MS).

## 2. Results and Discussion

In this study, we will describe the selection of the most efficient antagonist against SRP. The structural characterization and quantification of the main active metabolite produced in its CFS were achieved. Finally, the bactericidal/bacteriostatic activities of the antagonist extract against *Dickeya solani* were evaluated and compared with those of the synthetic molecule.

### 2.1. Choice of the Main Active Antagonist

In order to find the active metabolite to control SRP, we tested the CFSs obtained from bacteria grown in TY (tryptone and yeast) extract [25], a liquid, non-selective rich media known to promote bacteria metabolite production [26]. The CFSs of the selected bacteria: *Bacillus simplex* BA2H3, *Pseudomonas brassicacearum* PP1-210F and PA1G7, *Pseudomonas* sp. PA14H7, *Pseudomonas fluorescens* PA3G8, and *Pseudomonas lactis* PA4C2 [18] were confronted to a broad range of SRP (Table 1).

The CFSs of PA14H7, PA1G7, and PP1-210 showed a bactericidal effect against *P. atrosepticum* P8-Me25a (Table 1). These three bacteria had already presented a large-scale inhibitory effect against SRP during direct confrontation in vitro tests [19]. However, the CFS of PA14H7 was the only one that displayed a bacteriostatic effect against the whole tested panel of SRP (Table 1). In light of these results, PA14H7 seemed to be the best candidate able to produce active metabolite(s) against a broad range of SRP and, so, to be a suitable biocontrol agent in our strategy of controlling blackleg disease.

### 2.2. Evaluation of CFS-PA14H7 Extraction Methods

Following the selection of the antagonist, the second step was to determine when the bacteria reached the highest cell density in order to maximize the chance of detecting and identifying potential metabolites. We carried out a kinetic experiment, which revealed that the bacterial growth reached the stationary phase after 48 h of PA14H7 incubation (Supplementary Materials Figure S1). This time of incubation was used for all the upcoming steps.

We first attempted to directly analyze the CFS-PA14H7 versus TY medium alone. Unfortunately, the complexity of the LC-MS chromatograms, partly due to the composition of the medium (Supplementary Materials Table S1), did not allow us to point out the major differences that were present (Supplementary Materials Figure S2). Thus, we subsequently designed different extractions with commonly used solvents (including chloroform and ethyl acetate at pH 2 and pH 7, respectively) in order to simplify the chromatogram analysis and to recover a maximum of metabolites. The LC-MS chromatograms of the aqueous phases (Supplementary Materials Figure S3) and organic phases (Supplementary Materials Figure S4) were deemed to be quite similar no matter the extraction method used. Consequently, we attempted to differentiate the efficient phases through performing bioassays. In vitro tests were performed to evaluate their bacteriostatic and bactericidal activity against a strain of *Dickeya solani*, a major damageable pathogen affecting potatoes (Table 2 and Supplementary Materials Table S2).

The absence of an antagonistic effect of these aqueous phases indicates that the active metabolites are only present in the organic phases, which have shown a similar efficiency whatever the solvent and pH used for their extraction.

### 2.3. Identification of the Active Metabolite(s)

#### 2.3.1. LC-MS Analysis

**Figure 1:**
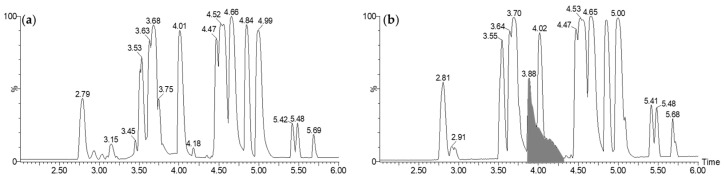
LC-MS (electrospray ionization in the positive ion mode: ESI^+^), base peak intensity (BPI) chromatograms of organic phases after extraction with chloroform at pH 7 of TY culture medium (**a**) and CFS-PA14H7 (**b**). The major difference observed between 3.80 min and 4.40 min has been highlighted.

To identify the active metabolite(s), a comparison between the LC-MS profiles of TY versus CFS-PA14H7 organic phases after extractions was carried out. For all extractions, the organic phase chromatograms highlighted a major difference corresponding to a very large peak diffusing from 3.80 min to 4.40 min, as exemplified for the chloroform extraction at pH 7 (Figure 1). We noted that this peak coeluted with another one at 4.01 min, which was also present in the TY extract.

**Figure 2:**
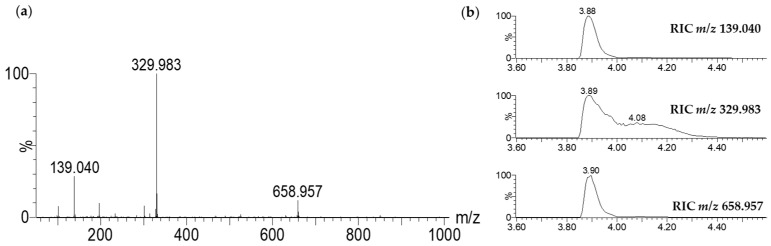
Mass spectrum of the large chromatographic peak observed between 3.80 min and 4.40 min on the LC-MS BPI trace of CFS-PA14H7 chloroform extract (**a**). The RIC of the ions observed at *m*/*z* 139.040, 329.983, and 658.957 are also presented (**b**).

The mass spectrum corresponding to this large peak has been presented in Figure 2a. It was mainly composed of three ions at *m*/*z* 139.040, 329.983, and 658.957. These ions could be attributed to the same organic molecule: alone ([M + H]^+^, C_7_H_6_O_3_, *m*/*z* 139.040), as a dimeric complex with iron ([2M − 2H + Fe]^+^, C_14_H_10_O_6_Fe, *m*/*z* 329.983) and its corresponding dimer generated under a high concentration in the gas phase (C_28_H_19_O_12_Fe_2_, *m*/*z* 658.957). This phenomenon has been evidenced by the reconstituted ionic current (RIC) traces of the different species presented in Figure 2b.

**Figure 3:**
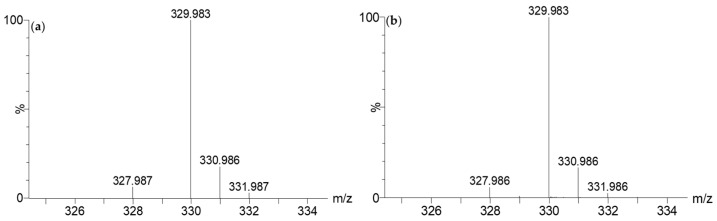
Theoretical isotopic pattern of the elemental composition of C_14_H_10_O_6_Fe (**a**) vs. the experimental isotopic pattern at *m*/*z* 329.983 (**b**).

The complex with iron was established through the characteristic isotopic pattern distribution of ^56^Fe/^54^Fe (Figure 3). The distorted aspect of its chromatographic peak (RIC, *m*/*z* 329.983, Figure 2b) was also specific to a complex, which is in equilibrium between its free and associated forms.

Specific research of the accurate mass (at *m*/*z* 139.040 and *m*/*z* 329.983, ±20 ppm) revealed that neither the molecule nor its iron complex were present in the TY extract, even under trace amounts (Supplementary Materials Figure S5).

Finally, using the LC-MS data (retention time, *m*/*z*), we carried out a non-targeting profiling approach in order to identify all the markers differentiating the culture medium TY from CFS-PA14H7. This analysis revealed that the iron complex (*m*/*z* 329.983) and the free molecule (*m*/*z* 139.040) were clearly the main abundant markers generated in the presence of PA14H7 (Supplementary Materials Table S3).

#### 2.3.2. Biologically Driven Purification Using Flash Chromatography

In order to separate and isolate the active molecule from the rest of the chromatogram, we carried out a flash chromatography on a 3 L chloroform pH 7 extraction of CFS-PA14H7. The evaporation of the solvent provided 156.22 mg of a brownish oil, which was purified on a C18 reverse phase column. The purification process was monitored using UV wavelengths at 244 nm and 320 nm. The latter corresponded to the two maximums of absorbance, recorded using the diode array detector (DAD), of the large peak eluting between 3.80–4.40 min during the LC-MS analysis (Figure 1 and Supplementary Materials Figure S6).

The flash chromatography fraction (F2) containing the peak detected by UV at 244 and 320 nm was collected (Supplementary Materials Figure S7), which formed a 7.5 mg yellow powder after evaporation. Fractions before (F1) and after (F3) the peak were also collected. The three fractions were biologically tested to confirm that the active metabolite was mainly present in F2. Fraction F1 did not display any biological activity, contrary to fraction F3, which exhibited a reduced bactericidal/bacteriostatic effect, probably due to the tailing of the peak collected in F2 (Table 3).

#### 2.3.3. Determination of the Metabolite Structure

**Figure 4:**
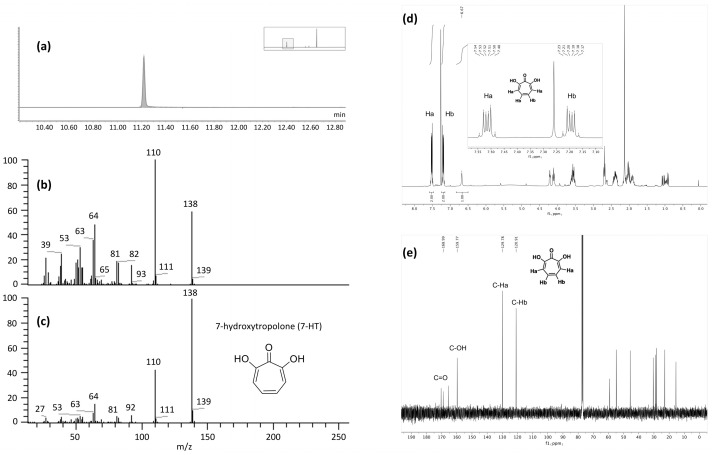
Analysis of the purified fraction F2 of CFS-PA14H7 after extraction (chloroform pH 7) and flash chromatography with GC-MS. (**a**) EI total ionic current (TIC) trace, (**b**) EI spectrum compared to the (**c**) EI spectrum of 7-HT from the NIST library, (**d**) ^1^H NMR and (**e**) ^13^C NMR spectra recorded in CDCl_3_.

The fraction F2 obtained after flash chromatography was subjected to different analytical techniques. Its LC-MS chromatogram and the corresponding mass spectrum confirmed that the main product, which was isolated, corresponds to the iron complex previously described in Section 2.3.1 (Supplementary Materials Figure S8). For unambiguous identification of the organic part of the complex, a GC-MS analysis was performed (Figure 4a).

Indeed, the temperatures used for GC injection and separation led to the dissociation of the complex in the gas phase. The electronic ionization low-resolution mass spectrum (EI, Figure 4b) pointed out a molecular ion M^+^**·** at *m*/*z* 138 in accordance with the [M + H]^+^ at *m*/*z* 139.040 observed in the electrospray ionization high-resolution mass spectrum (ESI-HRMS). The NIST library search led to two structure hypotheses: 7-hydroxytropolone (7-HT) or 2-ethoxyphenol. Only the elemental composition of 7-HT (C_7_H_6_O_3_) was compatible with the accurate mass measurement results obtained in ESI^+^ (C_7_H_6_O_3_ exp. *m*/*z* 139.0396 vs. th. *m*/*z* 139.0395 and C_7_H_5_O_3_FeC_7_H_5_O_3_ exp. *m*/*z* 329.9827 vs. th. *m*/*z* 329.9828). The EI spectrum of 7-HT derived from the NIST library is presented in Figure 4c and those of the other hypothesis has been displayed in the Supplementary Materials (Figure S9). In addition, we carried out ^1^H and ^13^C NMR experiments of F2 in CDCl_3_ (Figure 4d,e and Supplementary Materials Figure S10). Despite the presence of co-eluted impurities that were also detected in LC-MS and GC-MS, the chemical shifts of the main compound were attributed to the 7-HT structure.

### 2.4. Validation of 7-HT Structure and Activity

#### 2.4.1. Organic Synthesis of 7-HT

**Figure 5:**
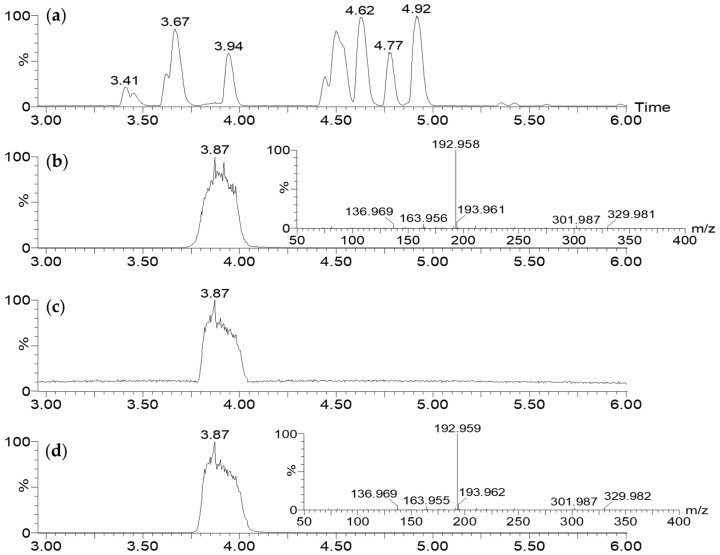
LC-MS/MS (ESI^+^) analysis of CFS-PA14H7 extracted with chloroform at pH 7: BPI (**a**) and RIC of *m*/*z* 329.983 with its corresponding MS/MS (30 eV) spectrum (**b**), compared with those of 7-HT obtained via synthesis (**c**,**d**).

7-HT is not commercially available, and in order to unambiguously confirm its structure, ability to form an iron complex, and activity, we carried out its synthesis in four steps starting from commercial tropolone, as previously described [27,28] (Supplementary Materials Figure S11). In this process, 7.5 g of 7-HT was obtained from 25 g of tropolone corresponding to a yield of 27%. NMR (Supplementary Materials Figure S12) and GC-MS (Supplementary Materials Figure S13) of the recrystallized synthetic 7-HT were in accordance with those obtained previously for the purified fraction F2. Nevertheless, on the ^1^H NMR spectrum of the purified extract (Figure 4d), we observed a broad singlet at 6.67 ppm integrating for one proton for the hydroxyl groups of the tropolone moiety. For the synthetic 7-HT, this singlet was shifted at 8.33 ppm and integrated for two protons. This phenomenon seems to indicate that 7-HT was present as a complex in CFS-PA14H7. An additional proof was obtained by injecting in LC-MS the synthetized 7-HT. As observed for the CFS-PA14H7 extracts, 7-HT was detected as an iron complex at the same retention time and with identical MS/MS fragments ions, confirming that the entities we observed were the same (Figure 5).

**Figure 6:**
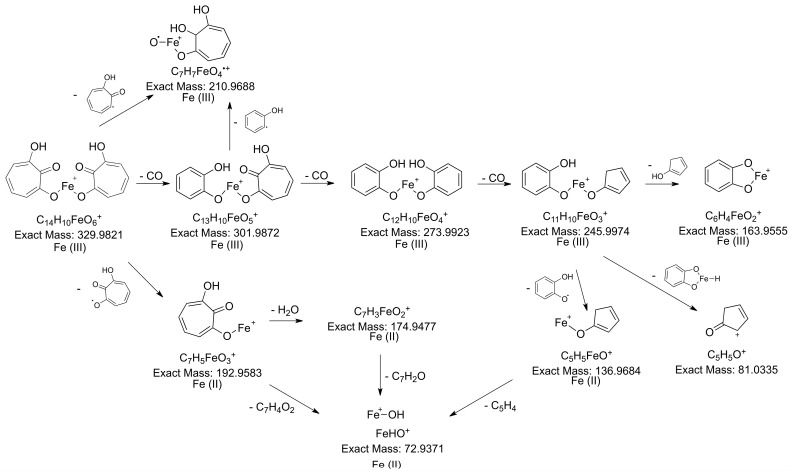
Proposed structure for the 7-HT:iron(III) 2:1 complex and its hypothetical fragmentation pattern obtained via MS/MS (30 eV).

The spectral data obtained via NMR and LC-MS indicate that 7-HT produced by PA14H7 forms a complex with iron. The positive ion generated in ESI^+^ (*m*/*z* 329.983, Figure 3) revealed a 7-HT:iron(III) complex with a stoichiometric ratio of 2:1. A proposal structure for this complex and its MS/MS fragmentation pattern is depicted in Figure 6 and Supplementary Materials Figure S14. The dimeric iron complex ion of 7-HT at *m*/*z* 329.982 gave rise after three successive losses of CO to the respective ions at *m*/*z* 301.987, 273.992, (very low intensity) and 245.997, which is consistent with the presence of carbonyl and hydroxyl groups on the 7-HT structure. Then, the fragment ion at *m*/*z* 245.997 underwent fragmentation to form three ions at *m*/*z* 163.955, 136.968, and 81.033. The ion at *m*/*z* 163.955 could be explained by a catechol structure complexed with Fe(III) by losing from *m*/*z* 245.997 a hydroxy-cyclopentadiene molecule. The fragment observed at *m*/*z* 136.968 could have come from the ion at *m*/*z* 245.997 through the loss of a catechol radical to give rise to an hydroxy-cyclopentadiene structure complexed by Fe(II). The last of the three ions produced from *m*/*z* 245.997 at *m*/*z* 81.033 could be explained by the loss of a catechol structure complexed with Fe(III). Afterwards, the fragment ion observed at *m*/*z* 210.968 could be obtained by two losses, either a tropolone radical from the precursor ion at *m*/*z* 329.982 or a phenol radical from the fragment at *m*/*z* 301.987, to give to the same monomeric radical ion of a 7-HT derivative that is always complexed by iron. The most abundant fragment ion at *m*/*z* 192.958 can be directly obtained from the precursor ion at *m*/*z* 329.982 through the loss of a hydroxy-tropolone radical, leading to a monomeric hydroxy-tropolone ion with Fe(II). This behavior towards fragmentation directly evidences the dimeric nature of this complex as a homodimer. It should be noted that this hydroxy-tropolone radical loss involved a change in the oxidation state of iron with a reduction from Fe(III) to Fe(II). A loss of H_2_O was observed from *m*/*z* 192.958 to form the ion at *m*/*z* 174.947. Finally, the hydroxide anion complexed by Fe(II) at *m*/*z* 72.938 could have potentially come from the ions at *m*/*z* 192.958, 174.947, and 136.968. However, at this stage, we were not able to determine the nature of the third ligand. One hypothesis could be another 7-HT molecule, as described for ferric tropolone, which was characterized as a trimer of the tropolone unit using single crystal X-ray diffraction [29], but other anions (sulfate, chloride, phosphate, etc.) coming from the growing medium are also possible.

#### 2.4.2. Quantification of 7-HT Produced by PA14H7

At this stage, we have clearly identified 7-HT as the main active metabolite produced by PA14H7 under TY growth-mediated conditions. This result was in accordance with previously published results showing that 7-HT was already observed and identified as an iron chelator in different *Pseudomonas* strains, such as *P. donguensis*, *P. qingdaonensis*, and *P. wadenswilerensis* [24,30]. This natural molecule is well known for its antibacterial and antifungal activities [31]. An important aspect was the possibility to accurately determine the amount produced by PA14H7, especially since 7-HT was complexed with iron in the culture medium.

The classical approach consists of a direct measurement of the UV absorbance at 327 nm in the CFS. The CFS-PA14H7 used for the determination of the amount of 7-HT produced by the bacteria revealed an OD_327nm_ of 1.02 and, consequently, a 14.2 mg/L concentration of 7-HT (Supplementary Materials Figure S15). This result corresponded to specific growth conditions (initial concentration of bacteria, medium volume, and incubation time). Indeed, when varying these conditions, we observed the OD_327nm_ ranging from 0.5 to 4 (corresponding to a 7-HT concentration from 7 mg/L to 55 mg/L). This was deemed to be in accordance with the results presented in the literature, where 7-HT concentration values between 30 mg/L and 90 mg/L were determined using other bacterial strains [24,30]. This method is easy to implement and can be directly used on the CFS; however, it is not specific to 7-HT and can lead to an over-estimation due to the UV absorbance of other metabolites produced by the bacteria.

Thus, we decided to complete direct UV measurements with quantifications of CFS-PA14H7 extracts through employing the techniques of LC-UV, LC-MS, and GC-MS, which allowed for the separation of the 7-HT molecule from other species. The objective was to compare with the previous result (14.2 mg/L) and also to determine the level of recovery of the different extraction conditions. For each analytical method, the CFS-PA14H7 extracts were quantified using a calibration curve generated with the synthetic 7-HT (Supplementary Materials Figure S16); the results are listed in Table 4. Globally, concentrations of 7-HT were deemed to be similar whatever the method used. Nevertheless, we found that recovery with ethyl acetate extraction at pH 7 was always lower than with other extraction methods, even though we ensured that no 7-HT residue was detected in the aqueous phase.

Regarding these results, all extraction conditions, except for ethyl acetate at pH 7, were acceptable for a good recovery of 7-HT in the CFS. A value around 9 mg/L was determined, when averaging the three techniques, which is not so far from the direct UV measurement (14.2 mg/L). As a reference, synthesized 7-HT was diluted in water at 9.6 mg/L and extracted with chloroform at pH 7 and ethyl acetate at pH 2. The result depicted in Table 4 revealed that the extraction of 7-HT in the water solution was complete. Concerning the analytical methods, GC-MS was the only technique which detected 7-HT as a non-iron complex molecule, and is therefore the most suitable and reliable for integration/quantification due to a better chromatographic resolution, leading to a sharper chromatographic peak than those observed for the complexed form in LC-UV and LC-MS.

#### 2.4.3. In Vitro Test of CFS-PA14H7 vs. Synthetic 7-HT

**Figure 7:**
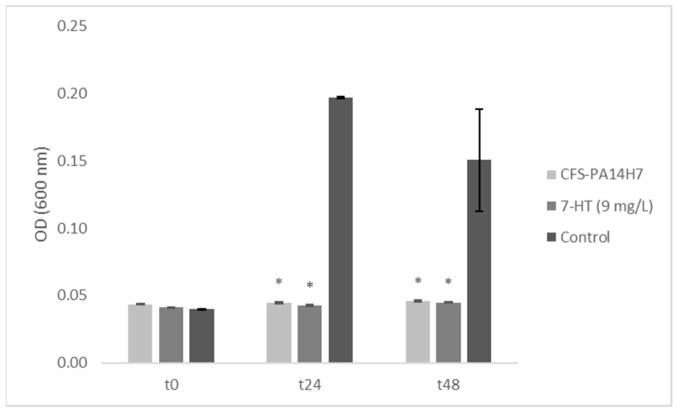
*Dickeya solani* growth in TY media containing CFS-PA14H7, 7-HT, or water (control) measured using optical density. Bars are the mean value for three biological assays and the standard errors are represented. (*) Significant difference between the mean values compared to the control according to the Kruskal–Wallis test at *p* < 0.05.

To confirm that 7-HT is closely linked to the biological activity of CFS-PA14H7, it was necessary to establish the ability of the synthetic molecule alone to inhibit the growth of *D. solani*. In vitro biological tests conducted in liquid medium at the concentration determined previously (9 mg/L) showed a similar growth-inhibitory effect of *D. solani* than CFS-PA14H7 (Figure 7).

In order to verify whether the observed activities on *D. solani* were bacteriostatic or bactericidal, the liquid formed as a result of its incubation was transferred into TY agar Petri dishes. The growth of *D. solani* colonies for the two tested conditions showed that only either 7-HT or CFS-PA14H7 had a bacteriostatic effect. Moreover, a preliminary in vivo assay conducted under controlled conditions showed a decrease in blackleg symptoms on potato plants. Indeed, we observed 30% of plants from the positive control expressing blackleg symptoms, compared to 20% from the 7-HT water solution that was added at 1 mg/L and 0% when the 7-HT water solution was added at 10 mg/L. Therefore, these findings indicate that a total inhibition of blackleg symptoms was observed for plants treated with the 7-HT solution at the dose corresponding to the concentration measured in the CFS of PA14H7. These results, along with those from the in vitro and in vivo tests, provide further proof that 7-HT, identified in CFS-PA14H7 as an iron complex, is involved in antagonistic activity.

## 3. Materials and Methods

**Figure 8:**
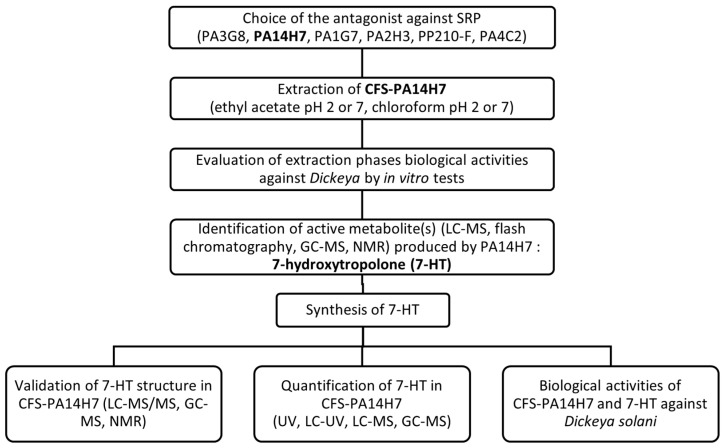
Description of the different steps leading to the selection of an efficient antagonist against SRP—the causal agents of potato blackleg—along with the identification, quantification, and evaluation of the biological activity of the main metabolite produced by this antagonist.

The flowchart of the experimental protocol used in this study is depicted in Figure 8.

### 3.1. Materials

For the biological test, bacto tryptone and bacto yeast extract were provided by Fisher Scientific (Illkirch, France). Potassium phosphate monobasic, potassium phosphate dibasic, and agar were purchased from Sigma-Aldrich (Saint-Quentin, Fallavier, France).

For extraction, purification, and chemical analysis, chloroform, ethyl acetate, methanol, formic acid, and water (of ULC-MS grade) were all purchased from Biosolve BV (Valkenswaard, The Netherlands). Hydrochloric acid solution (1M) was purchased from Sigma-Aldrich (Saint-Quentin, Fallavier, France). Deuterated reagent, such as deuterated chloroform (CDCl_3_), was purchased from Euriso-top (Saint-Aubin, France).

For organic synthesis, tropolone, 18-crown-6, iodomethane, N-bromosuccinimide, trifluoroacetic acid, and acetic anhydride were purchased form Thermo Scientific (Illkirch, France). Benzene and toluene were purchased from Sigma-Aldrich (Saint-Quentin, Fallavier, France).

### 3.2. Biological Material

*Bacillus simplex* BA2H3, *Pseudomonas brassicacearum* PP1-210F and PA1G7, *Pseudomonas* PA14H7, *Pseudomonas fluorescens* PA3G8, and *Pseudomonas lactis* PA4C2 were previously isolated from potato soil rhizosphere [18,32]. *Dickeya solani* 3337 [33], *P. atrosepticum* P8-Me25a, *P. parmentieri* P13-CH22, *P. brasiliense* P1-15C1, *P. polaris* P1-10C1, *D. dianthicola* P15-29, and *D. solani* P5-Sp1a were previously isolated from potato blackleg symptoms. All bacteria strains were stored at −80 °C in 25% glycerol for long-time conservation. Prior to their utilization, bacterial strains were always revived on Petri dishes with TY agar. All bacterial cultures were inoculated at 1/1000 of their volume with a preculture of one colony into 5 mL of TY and incubated at 27 °C under agitation (130 rpm) for overnight to 48 h depending on the volume (ranging from 20 mL to 1 L). For antagonistic strains, their supernatants were obtained after 15 min centrifugation at 10,000× *g* and a filtration on a 0.2 µm filter of the bacterial culture.

### 3.3. Biological Test

Biological tests were conducted in 96 microplate wells; these were filled with 80 µL of TY medium culture. Next, 100 µL of filtered supernatant or synthesized molecule solution, at a known concentration, was added and mixed into the TY medium by pipetting. All solutions tested were realized in triplicate. The solution was replaced by sterilized water for the blank. Finally, 20 µL of a solution of *Dickeya solani* 3337 adjusted at 10^6^ CFU/mL were inoculated. Bacteriostatic effects were observed by comparing the OD_600nm_ at 0 h, 24 h, and 48 h of incubation at 27 °C and 130 rpm to the control condition. The bacterial concentration was first estimated through measuring the OD at 600 nm with a Multiskan™ GO Microplate Spectrophotometer (Thermo Scientific, Illkirch, France). Where no bacterial growth was observed after 48 h, the mix was spotted on TY agar Petri dishes and incubated at 27 °C. If colonies appeared after 48 h, the CFS was determined to exhibit a bacteriostatic effect; if not, the CFS was deemed to exert a bactericidal effect.

### 3.4. Kinetics Growth

A growth kinetics was realized on 1L of culture PA14H7 in TY medium incubated at 27 °C and under 130 rpm agitation. Each hour, 1 mL of medium was collected and the OD at 600 nm was measured using multiskango^®^ (Thermo Scientific, Illkirch, France). For the control, 1 mL of TY medium free from inoculum was used.

### 3.5. Extraction Method of CFS-PA14H7

As previously described in Section 3.2, 300 mL of CFS-PA14H7 was prepared. For extraction at pH 2, the pH was adjusted through the addition of a solution of HCl (1M). The cell-free supernatant was then extracted with 3 × 100 mL of organic solvent (ethyl acetate or chloroform). Organic and aqueous phases were rotary evaporated. The organic resulting extract was resuspended in 10 mL of methanol, and the aqueous phase was diluted in 10 mL of water.

### 3.6. LC-UV-MS

Separations were performed using an ACQUITY UPLC H-Class (Milford, MA, USA) system coupled on-line with an ACQUITY PDA detector and a Synapt G2-Si QTOF high-resolution hybrid mass spectrometer, equipped with an electrospray ionization (ESI) source (Z-spray) and an additional sprayer (Lock Spray) for the reference compound (Waters, Manchester, UK). The CFS-PA14H7 extracts and 7-HT standard were separated on an Acquity UPLC CSH C18 (1.7 µ 100 mm × 2.1 mm) chromatography column (Waters) maintained at 50 °C. The elution was performed using a 0.4 mL/min mobile phase gradient of water (A) and methanol (B), which both contained 0.1% formic acid, and was programmed as follows (A:B): 90:10 (t = 0 min), 90:10 (t = 1 min), 10:90 (t = 7 min), 10:90 (t = 12 min), 90:10 (t = 13 min), and 90:10 (t = 18 min). Most of the time, 0.2 µL of each sample was injected. The UV acquisitions were performed using an PDA detector ranging from 190 to 400 nm. The 7-HT signal was processed at 320 nm for quantification. The ESI source was operated in the positive ionization mode (ESI^+^) using a capillary voltage of 3 kV. The following ESI source conditions were: sampling cone voltage, 40 V; source offset, 40 V; source temperature, 120 °C; desolvation gas temperature, 450 °C; desolvation gas flow, 800 L/h; and cone gas flow, 50 L/h. Nitrogen (>99.5%) was employed as the desolvation and cone gas. Mass calibration was carried out using a sodium formate solution (10mM NaOH in isopropanol/water/formic acid 49.9:49.9:0.2) and Leu-enkephalin (*m*/*z* 556.2771) was used as the lock mass solution for accurate measurements (1 ng/µL in H_2_O/CH_3_CN/formic acid 50:49.9:0.1). The scan range was 50–2000 at 0.2 s/scan. The TOF was operated in the resolution mode (resolution FWHM: 25,000). The HRMS spectra were recorded in the centroid mode. For quantification, the reconstructed ionic current of the 7-HT iron complex ion (*m*/*z* 329.983) was integrated (window of 50 ppm). For structural confirmation, MS/MS experiments were performed using a trap cell collision energy of 30 eV with argon (99.999%) as the collision gas. Data acquisition and processing were performed using MassLynx software (V4.2, Waters).

### 3.7. Purification by Flash Chromatography

For this experiment, 3 L of CFS-PA14H7 was extracted with chloroform (3 × 1 L) at pH 7. After evaporation of the organic phase, the residue was dissolved in 2.5 mL of methanol/water solution (80/20) and subjected to flash chromatography separation on a Reveleris^®^ PREP Purification System equipped with a FP ECOFLEX C18 12 g column (BUCHI, Villebon-sur-Yvette, France). The elution was performed using a 30 mL/min mobile phase gradient of water (A) and methanol (B), which was programmed as follows (A:B): 98:2 (t = 0 min), 98:2 (t = 2 min), 0:100 (t = 30 min), and 0:100 (t = 2 min). The detection was recorded using UV and an evaporative light scattering detector (ELSD). The fractions of interest were collected using two wavelengths: 244 nm and 320 nm.

### 3.8. GC-MS

The CFS-PA14H7 extracts and 7-HT solutions prepared for the LC-UV-MS analyses were also monitored via GC-MS using a TRACE 1300 gas chromatograph coupled with an ISQ 7000 single quadrupole mass spectrometer (Thermo Scientific, San Jose, CA, USA). For this procedure, 1 µL of each sample was injected using a 1:25 split ratio and an injector temperature of 230 °C. The temperature gradient started at 70 °C (5 min) and was then was increased at a speed of 15 °C/min for 23 min until reaching 310 °C (2 min). The column used for gas chromatography separation was a TR-5MS (length: 30 m; inner diameter: 0.25 mm; and film thickness: 0.25 µm) from Thermo Scientific. Helium was used as carrier gas at a flow rate of 1 mL/min. The electron impact (EI) ionization mode at 70 eV was used with the source temperature set to 280 °C. The mass spectra were recorded after a solvent delay of 5.5 min using a mass range of 50 to 650 *m*/*z* at a rate of 2 scan/s. For identification of the 7-HT, the NIST library was used. For quantification, the reconstructed iron current of the molecular ion of 7-HT (M^+^**·** *m*/*z* 138) was integrated and validated with a presence of the 7-HT fragment at *m*/*z* 110. Acquisition and processing were performed with Chromeleon software, Version 7.2.

### 3.9. NMR

^1^H and ^13^C nuclear magnetic resonance (NMR) spectra were recorded on an AVANCE III HD 400 MHz spectrometer (Brucker, Wissembourg, France) at 400 and 100 MHz, respectively. Chemical shifts were reported in parts per million (ppm) and spectra were calibrated using the non-deuterated residual solvent peak for CDCl_3_ (^1^H: δ = 7.26 ppm, and ^13^C: δ = 77.16 ppm). Peak multiplicity was reported as: singlet (s), doublet (d), doublet of doublet (dd), and broad (br). Acquisition was controlled with TopSpin 3.6.2 and the spectra were processed with Mestrenova 14.3.1. The spectra of 7-HT obtained via synthesis was used as a reference and compared to the CFS-PA14H7 spectra after extraction and purification (Supplementary Materials Figure S12). The detail of the 7-HT spectra was as follows: ^1^H NMR (CDCl_3_, 25 °C, 400 MHz): *δ_H_* 8.33 (br s, 2H), 7.51 (dd, *J* = 7.4, 3.8 Hz, 2H), 7.18 (dd, *J* = 7.4, 3.8 Hz, 2H). ^13^C NMR (CDCl_3_, 25 °C, 100 MHz): *δ_C_* 168.9 (C), 159.9 (2 × C), 129.8 (2 × CH_2_), and 121.0 (2 × CH_2_).

### 3.10. Quantification of 7-HT

Calibration solutions of 7-HT were prepared using successive dilutions starting from a stock solution of synthesized 7-HT in methanol. The concentration range was established between 0.07 mg/mL and 0.56 mg/mL. Fresh mother solution and concentration range were prepared for each new series of measurements. For direct UV quantification of the CFS-PA14H7, and to maintain the similar conditions of measurement, the successive dilutions of 7-HT stock solution were realized in the growing TY medium. Firstly, UV spectra was recorded from 200 to 800 nm of synthesized 7-HT and CFS-PA14H7 with a Multiskan™ GO Microplate Spectrophotometer (Thermo Scientific, Illkirch, France). For concentration measurements, we selected the wavelength at 327 nm corresponding to the maximum of 7-HT absorbance. For the other quantification results obtained using the hyphenated analytical methods (LC-UV, LC-MS, and GC-MS), the data processing method has been detailed in the Materials and Methods section related to the corresponding technique.

### 3.11. Preliminary In Vivo Assay

The preliminary in vivo assay was conducted as a “potato/*D. solani* pathosystem” under controlled conditions (16 h day at 22 °C and 8 h night at 14 °C). For each modality, 10 potato tubers free from contamination, >28 mm in size, and from the Agria variety, were planted individually in 2 L pots filled with commercial substrate (Agaris Professional, Agaris, Gand, Belgium). Plantation was the reference for t_0_, and 100 mL of 7-HT water solutions at 1 mg/L or 10 mg/L were added to the plants 4 times at t_3weeks_, t_4weeks_, t_6weeks_, and t_8weeks_. Inoculation was carried out once all the plants fully emerged, that corresponded to t_3.5weeks_, by watering each plant with 100 mL of *D. solani* 3337 water solution adjusted at 10^7^ CFU/mL. Plants only inoculated with *D. solani* corresponding to the positive control were used for the comparison with modalities inoculated and treated with 7-HT solutions. For the negative control, potato plants were only watered with water. Each two weeks after inoculation, a fertilization was carried out with a N-P-K (17-5-19) Algospeed^®^ water solution (CompoExpert^®^, Levallois-Perret, France). Visual appearance of blackleg symptoms of the aerial part during vegetation was noted three times per week until t_10weeks_.

## 4. Conclusions

We identified that the cell-free supernatant of PA14H7 has potent antagonistic activity against soft rot *Pectobacteriaceae*. We demonstrated, using the respective benefits of analytical techniques (LC-MS, GC-MS, and NMR) and of in vitro and in vivo biological tests, that this activity was linked to the production in the CFS of the 7-HT molecule complexed with iron. Quantification of 7-HT was achieved through direct UV measurements in the CFS, but also after a specific chromatographic separation using LC-UV, LC-MS, and GC-MS in the CFS extracts. GC-MS was determined to be the most reliable technique due to its higher chromatographic resolution. A detailed MS/MS study has shown the dimeric nature of this complex as a homodimer and has allowed to establish its structure and stoichiometry as a 7-HT:iron(III) 2:1 complex. 7-HT has already been described in other *Pseudomonas* species, such as *P. donguensis* [30], and its ability to complex iron could be involved in the antagonistic activity against SRP by limiting its access to iron for the development of SRP. However, the comprehension of the mechanism of action of 7-HT needs further investigation. The cluster gene of 7-HT biosynthesis has been identified in *P. qingdaonensis* and *P. wadenswilerensis* [24]. In this manner, the construction of a mutant deficient in the 7-HT biosynthesis pathways will allow us to clearly establish whether the antagonistic activity is only due to this metabolite and not due to the presence of other large molecules, such as peptides and proteins. It will also make it possible to exclude other mechanisms, such as spatial competition, as well as synergic effects with other metabolites. Finally, the mutant will help to determine the essential precursor of 7-HT biosynthesis [34] in order to increase 7-HT bioproduction by modulating the growing media compositions. Indeed, it is known that the medium can affect metabolite production [35].

To limit pathogen propagation in general and the blackleg disease in particular, the use of the biocontrol approach faced multiple issues. The successful transition from an in vitro or greenhouse experiment to field conditions are not often conclusive due to the annual variation of climatic conditions, as humidity or temperature can affect the survival and multiplication of the biocontrol agent [36]. In order to reduce these abiotic influences, the use of active metabolites in addition to the biocontrol agent could be a possible solution [21]. In this context, it could be useful to develop a greener and faster synthesis of 7-HT, which is not marketed. The use of commercial analogous molecules that are able to form an iron complex could also be investigated.

## Figures and Tables

**Table 1:** Antagonistic effect of the selected bacteria cell-free supernatants (CFS) on the growth of soft rot *Pectobacteriaceae* (SRP) from the genera *Pectobacterium* and *Dickeya*.

	CFS of Antagonistic Bacteria Strain						
SRP Strain	Blank	PA3G8	PA1G7	PA4C2	PA14H7	PA2H3	PP1-210F
*P. atrosepticum* P8-Me25a	−	−	++	−	++	−	++
*P. parmentieri* P13-CH22	−	−	−	−	+	−	−
*P. brasiliense* P1-15C1	−	−	−	−	+	−	−
*P. polaris* P1-10C1	−	−	−	−	+	−	−
*D. dianthicola* P15-29	−	−	−	−	+	−	−
*D. solani* P5-Sp1a	−	−	−	−	+	−	−
*D. solani* 3337	−	−	−	−	+	−	−

The effect of the CFS of the antagonists PA3G8, PA1G7, PA4C2, PA14H7 PA2H3, and PP1-210F is marked “−” if no growth inhibition of pectinolytic bacteria was observed, “+” for bacteriostatic effect, and “++” for bactericidal effect. Blank correspond to culture of SRP strains in TY medium alone.

**Table 2:** Comparison of biological activity against *Dickeya solani* 3337 of the aqueous and organic phases obtained from the different extraction conditions of CFS-PA14H7.

		Aqueous Phase (Dilution Factor)						Organic Phase (Dilution Factor)					
Extraction Condition		1	0.5	0.25	0.1	0.05	0.01	1	0.5	0.25	0.1	0.05	0.01
Ethyl acetate	pH 2	−	−	−	−	−	−	++	++	++	++	++	−
	pH 7	−	−	−	−	−	−	++	++	++	++	+	−
Chloroform	pH 2	−	−	−	−	−	−	++	++	++	++	++	−
	pH 7	−	−	−	−	−	−	++	++	+	+	+	−

“−” no effect, “+” for bacteriostatic effect, and “++” for bactericidal effect. A dilution factor of 1 corresponds to an extraction of 300 mL CFS-PA14H7 concentrated 30 times.

**Table 3:** Bacteriostatic/bactericidal effect against *Dickeya solani* 3337 of the flash chromatography fractions obtained after purification of a 3 L CFS-PA14H7 extraction.

	Dilution Factor							
Fraction	1	0.5	0.25	0.1	0.05	0.01	0.005	0.001
**F1**	−	−	−	−	−	−	−	−
**F2**	++	++	++	++	++	+	+	−
**F3**	++	++	+	−	−	−	−	−

“−” no effect, “+” for bacteriostatic effect, and “++” for bactericidal effect. Dilution 1 corresponds to the collected fractions.

**Table 4:** 7-HT measured concentrations in CFS-PA14H7 (mg/L) for each extraction condition, which were processed using LC-UV (λ 320 nm), LC-MS (*m*/*z* 329.983), and GC-MS (*m*/*z* 138).

			Analytical Method		
	Extraction Condition		LC-UV	LC-MS	GC-MS
**Extraction of** **CFS PA14H7 ^1^**	Ethyl acetate	pH 2	8.9	8.5	12.4
		pH 7	5.7	6.0	7.3
	Chloroform	pH 2	7.9	8.2	10.0
		pH 7	10.3	8.5	8.9
**Synthetic 7-HT in water (9.6 mg/L)**	Ethyl acetate	pH 2	n.d.	8.9	9.7
	Chloroform	pH 7	n.d.	11.3	9.2

^1^ Extraction and quantification were conducted on CFS-PA14H7 obtained from 1 L of PA14H7 culture in TY after 48 h incubation (values expressed in mg/L are a mean value obtained from three replicates). n.d.—not determined.

## Data Availability

The data presented in this study are available in Supplementary Material.

## Supplementary Materials

The following supporting information can be downloaded at: https://www.mdpi.com/article/10.3390/molecules28176207/s1, Figure S1. Growth kinetics of *Pseudomonas* PA14H7 in TY medium; Table S1. Detailed composition of bacto tryptone (named T); Figure S2. LC-MS (electrospray ionization in the positive ion mode: ESI^+^) and base peak intensity (BPI) chromatograms of TY culture medium (A) and CFS-PA14H7 (B); Figure S3. LC-MS (ESI^+^) comparison of TY culture medium (left) and CFS-PA14H7 (right) chromatograms of aqueous phases after extraction with chloroform at pH 7 (A), chloroform at pH 2 (B), ethyl acetate pH 7 (C), or ethyl acetate pH 2 (D); Figure S4. LC-MS (ESI^+^) comparison of TY culture medium (left) and CFS-PA14H7 (right) chromatograms of organic phases after extraction with chloroform at pH 7 (A), chloroform at pH 2 (B), ethyl acetate pH 7 (C), or ethyl acetate pH 2 (D). Extraction performed on the same batch of TY and PA14H7-filtered supernatants; Table S2: optical density at 600 nm (OD 600 nm) measurement of organic and aqueous phases of CFS-PA14H7 extract with chloroform or ethyl acetate at pH 2 or pH 7 after 0 h, 24 h, and 48 h of incubation with *D. solani*; Figure S5: the reconstituted ion current (RIC) of the ions observed between 3.6 and 4.6 min at *m*/*z* 139.040 (A), *m*/*z* 329.983 (B), and *m*/*z* 658.957 (C) in TY culture medium (left) and CFS-PA14H7 (right); Table S3. Summary of markers present in CFS-PA14H7 and absent in TY medium analyzed with the marker lynx (Waters, Masslynx V4.2); Figure S6. UV trace corresponding to the maximum of absorbance of the iron complex (B), absent in TY UV trace (A). Corresponding LC-MS (ESI^+^) BPI chromatograms for the TY culture medium (C) and CFS-PA14H7 (D), highlighting the presence of the complex at *m*/*z* 329.983; Figure S7. Ultra-violet (UV) at 224 nm (blue) and 320 nm (purple) and evaporative light scattering diffusion (ELSD) (green) chromatograms of the flash chromatography purification of CFS-PA14H7 using a C18 column. Fraction F1 is highlighted in green, F2 in orange, and F3 in blue; Figure S8. LC-MS (ESI^+^) chromatograms of fraction F2 resulting from CFS-PA14H7 purification by flash chromatography, BPI trace (A), and RIC of the ions at *m*/*z* 139.040 (B) and *m*/*z* 329.983 (C). The purified iron complex is effectively detected between 3.80 and 4.40 min. The corresponding mass spectra are also depicted; Figure S9. EI NIST library spectra of the different hypotheses for the molecular ion observed at *m*/*z* 138. 7-HT (A), 2-ethoxyphenol and isomers (B); Figure S10: Detailed NMR spectra of fraction F2 resulting from CFS-PA14H7 purification using flash chromatography: (A) ^1^H NMR (400 MHz) and (B) ^13^C NMR (100 MHz); Figure S11: synthesis of 7-hydroxytropolone starting from commercial tropolone; Figure S12: (A) ^1^H NMR and (B) ^13^C NMR spectra of 7-HT obtained via synthesis and (C) ^1^H NMR spectra and (D) ^13^C NMR spectra comparison of fraction F2 resulting from CFS-PA14H7 purification using flash chromatography (top) and synthetic 7-HT (bottom) between 6.3 and 9.1 ppm; Figure S13: GC-MS chromatograms and spectra of 7-HT obtained via synthesis (A, C) and of fraction F2 resulting from CFS-PA14H7 purification using flash chromatography (B, D); Figure S14: proposal fragmentation pattern of 7-HT iron complex ion (*m*/*z* 329.983) via MS/MS (30 eV); Figure S15: (a) UV spectrum of CFS-PA14H7 (1L, 48h) and of 7-HT of various concentrations in solution in TY (b) calibration curve of 7-HT in TY solution, at 327 nm ranging from 1.4 to 27.6 mg/L; and Figure S16: standard curve performed with the 7-HT synthetic molecule ranging from 0.07 mg/mL and 0.56 mg/mL according to LC-UV (320 nm) (A), LC-MS (*m*/*z* 329.983) (B), and GC-MS (*m*/*z* 138) analysis (C).
