# Supplementary material for: Pseudomonas PA14H7: Identification and Quantification of the 7-Hydroxytropolone Iron Complex as an Active Metabolite against Dickeya, the Causal Agent of Blackleg on the Potato Plant
Source: Molecules. 2023 Aug 23;28(17):6207. doi: 10.3390/molecules28176207 (PMC10488565; doi:10.3390/molecules28176207)
Supplement: Supplementary file 1 [file molecules-28-06207-s001.zip › molecules-2521076-supplementary.pdf]

Supplementary materials: *Pseudomonas* PA14H7: Identification and Quantification of the 7-Hydroxytropolone Iron Complex as an Active Metabolite against *Dickeya*, the Causal Agent of Blackleg on the Potato Plant

Summary

Figure S1: Growth kinetics of Pseudomonas PA14H7 in TY medium.

Table S1: Detailed composition of Bacto Tryptone (named T)

Figure S2: LC-MS (electrospray ionization in the positive ion mode: ESI^+), base peak intensity (BPI) chromatograms of TY culture medium (A) and CFS-PA14H7 (B)

Figure S3: LCMS (ESI^+) comparison of TY culture medium (left) and CFS-PA14H7 (right) chromatograms of aqueous phases after extraction with chloroform at pH 7 (A), chloroform at pH 2 (B), ethyl acetate pH 7 (C) or ethyl acetate pH 2 (D).

Figure S4: LCMS (ESI^+) comparison of TY culture medium (left) and CFS-PA14H7 (right) chromatograms of organic phases after extraction with chloroform at pH 7 (A), chloroform at pH 2 (B), ethyl acetate pH 7 (C) or ethyl acetate pH 2 (D). Extraction performed on the same batch of TY and PA14H7 filtered supernatant.

Table S2: Optical Density at 600 nm (OD 600nm) measurement of organic and aqueous phases of CFS-PA14H7 extract with chloroform or ethyl acetate at pH 2 or pH 7 after 0 h, 24 h and 48 h of incubation with *D. solani*.

Figure S5: The reconstituted ion current (RIC) of the ions observed between 3.6 and 4.6 min at m/z 139.040 (A), m/z 329.983 (B) and m/z 658.957 (C) in TY culture medium (left) and CFS-PA14H7 (right).

Table S3: Summary of markers present in CFS-PA14H7 and absent in TY medium analyzed with marker lynx (Waters, Masslynx V4.2).

Figure S6: UV trace corresponding to the maximum of absorbance of the iron complex (B), absent in TY UV trace (A). Corresponding LC-MS (ESI^+) BPI chromatograms for TY culture medium (C) and CFS-PA14H7 (D) highlighting the presence of the complex at m/z 329.983.

Figure S7: Ultra-Violet (UV) at 224 nm (blue) and 320 nm (purple) and Evaporative Light Scattering Diffusion (ELSD) (green) chromatograms of the flash chromatography purification of CFS-PA14H7 using a C18 column. Fraction F1 is highlighted in green, F2 in orange, and F3 in blue.

Figure S8: LC-MS (ESI+) chromatograms of fraction F2 resulting from CFS-PA14H7 purification by flash chromatography, BPI trace (A), RIC of the ions at m/z 139.040 (B) and m/z 329.983 (C). The purified iron complex is effectively detected between 3.80 and 4.40 min. The corresponding mass spectra are also depicted.

Figure S9: EI NIST library spectra of the different hypothesis for the molecular ion observed at m/z 138. 7-HT (A) and 2-ethoxyphenol and isomers (B).

Figure S10: Detailed NMR spectra of fraction F2 resulting from CFS-PA14H7 purification by flash chromatography: (A) ^1H NMR (400 MHz) and (B) ^{13}C NMR (100 MHz) .

Figure S11: Synthesis of 7-hydroxytropolone starting from commercial tropolone.

Figure S12: (A) ^{1}H NMR and (B) ^{13}C NMR spectra of 7-HT obtained by synthesis and (C) ^{1}H NMR spectra and (D) ^{13}C NMR spectra comparison of fraction F2 resulting from CFS-PA14H7 purification by flash chromatography (top) and synthetic 7-HT (bottom) between 6.3 and 9.1 ppm.

Figure S13 : GC-MS chromatograms and spectra of 7-HT obtained by synthesis (A, C) and of fraction F2 resulting from CFS-PA14H7 purification by flash chromatography (B, D).

Figure S14: Proposal fragmentation pattern of 7-HT iron complex ion (m/z 329.983) by MS/MS (30 eV).

Figure S15: (a) UV spectrum of CFS-PA14H7 (1L, 48h) and of 7-HT of various concentrations in solution in TY (b) calibration curve of 7-HT in TY solution, at 327 nm ranging from 1.4 to 27.6 mg/L.

Figure S16: Standard curve performed with 7-HT synthetic molecule range from 0.07 mg/mL and 0.56 mg/mL according to LC-UV (320nm) (A), LC-MS (m/z 329.983) (B) and GC-MS (m/z 138) analysis (C).

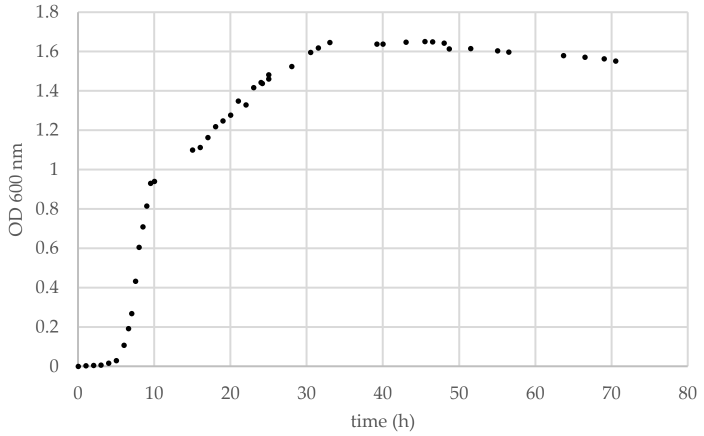

Figure S1: Growth kinetics of Pseudomonas PA14H7 in TY medium.

Table S1: Detailed composition of Bacto Tryptone (named T)

Total nitrogen (%)	Amino nitrogen (%)	AN/TN	Total carbohydrate (mg/g)	Ash (%)	Loss on drying (%)	NaCl (%)	pH (1% solution)	Calcium (µg/g)	Iron (µg/g)	Magnesium (µg/g)	Potassium (µg/g)	Sodium (µg/g)	Chloride (%)	Sulfate (%)	Phosphate (%)
13.3	5.3	0.4	4.3	6.6	2.3	0.0	7.3	256	23.0	195	3257	33910	0.06	0.33	2.58

Alanine (% free)	Arginine (% free)	Asparagine (% free)	Aspartic acid (% free)	Cystine (% free)	Glutamic acid (% free)	Glutamine (% free)	Glycine (% free)	Histidine (% free)	Isoleucine (% free)	Leucine (% free)	Lysine (% free)	Methionine (% free)	Phenylalanine (% free)	Proline (% free)	Serine (% free)	Threonine (% free)	Tryptophan (% free)	Tyrosine (% free)	Valine (% free)
1.0	2.2	0.6	0.4	0.3	1.4	0.1	0.2	0.5	1.3	4.8	5.5	1.0	3.0	0.2	0.7	0.7	0.8	0.5	1.7

Alanine (% total)	Arginine (% total)	Aspartic acid (% total)	Glutamic acid (% total)	Glycine (% total)	Histidine (% total)	Isoleucine (% total)	Leucine (% total)	Lysine (% total)	Methionine (% total)*	Phenylalanine (% total)	Proline (% total)	Serine (% total)*	Threonine (% total)	Tyrosine (% total)	Valine (% total)
3.2	5.0	5.2	15.1	1.7	1.9	5.5	7.5	6.2	2.1	5.2	6.6	2.2	1.8	1.3	5.9

Legend

□ Free amino acids □ Total amino acids

0.0 Below limit of detection

*Composition of Bacto Yeast extract (named Y) was not available from the provider.

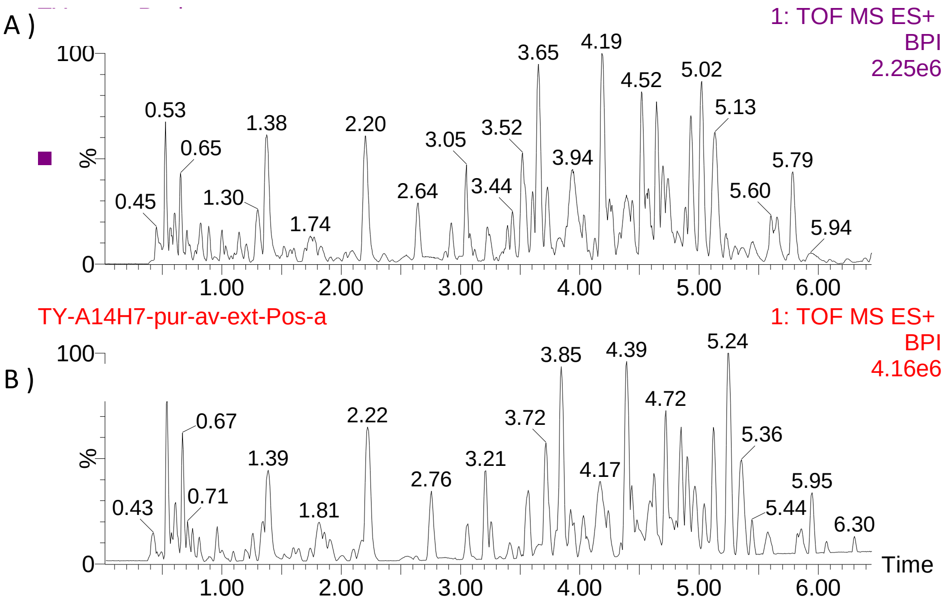

Figure S2: LC-MS (electrospray ionization in the positive ion mode: ESI+), base peak intensity (BPI) chromatograms of TY culture medium (A) and CFS-PA14H7 (B)

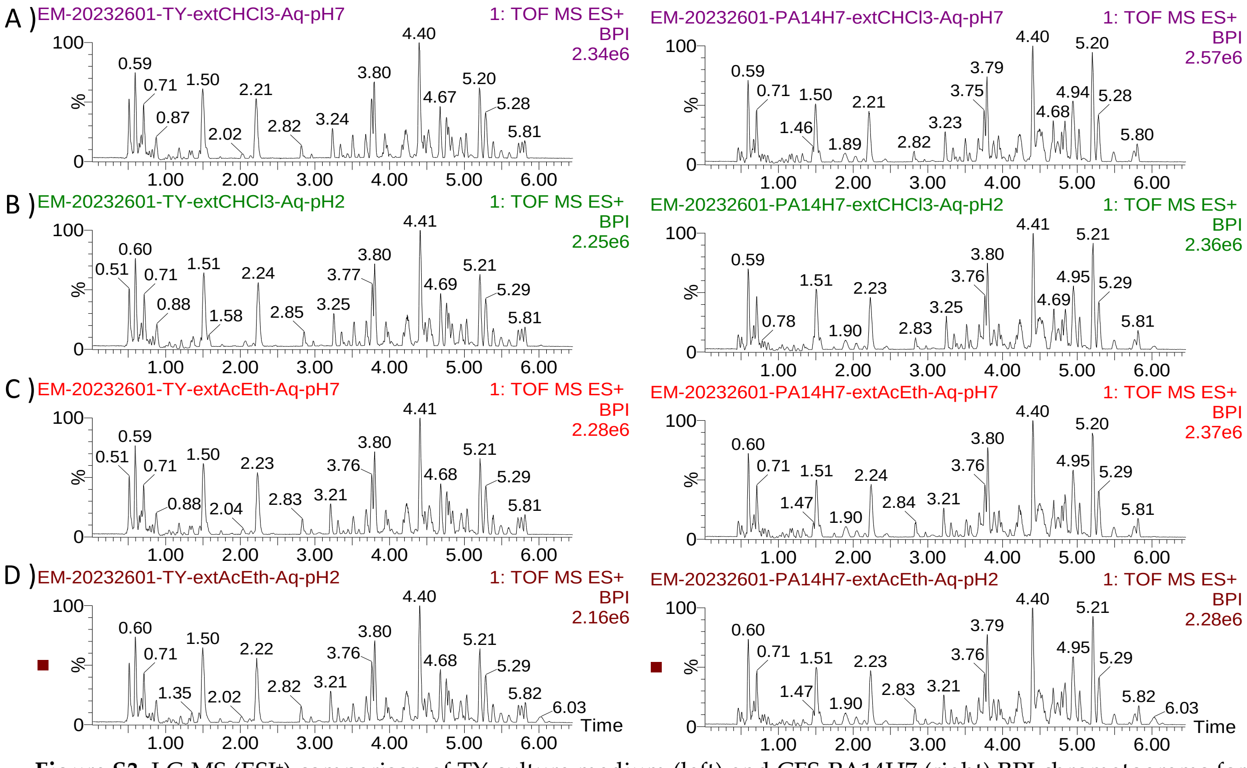

Figure S3: LC-MS (ESI+) comparison of TY culture medium (left) and CFS-PA14H7 (right) BPI chromatograms for aqueous phases after extraction with chloroform at pH 7 (A), chloroform at pH 2 (B), ethyl acetate pH 7 (C) or ethyl acetate pH 2 (D).

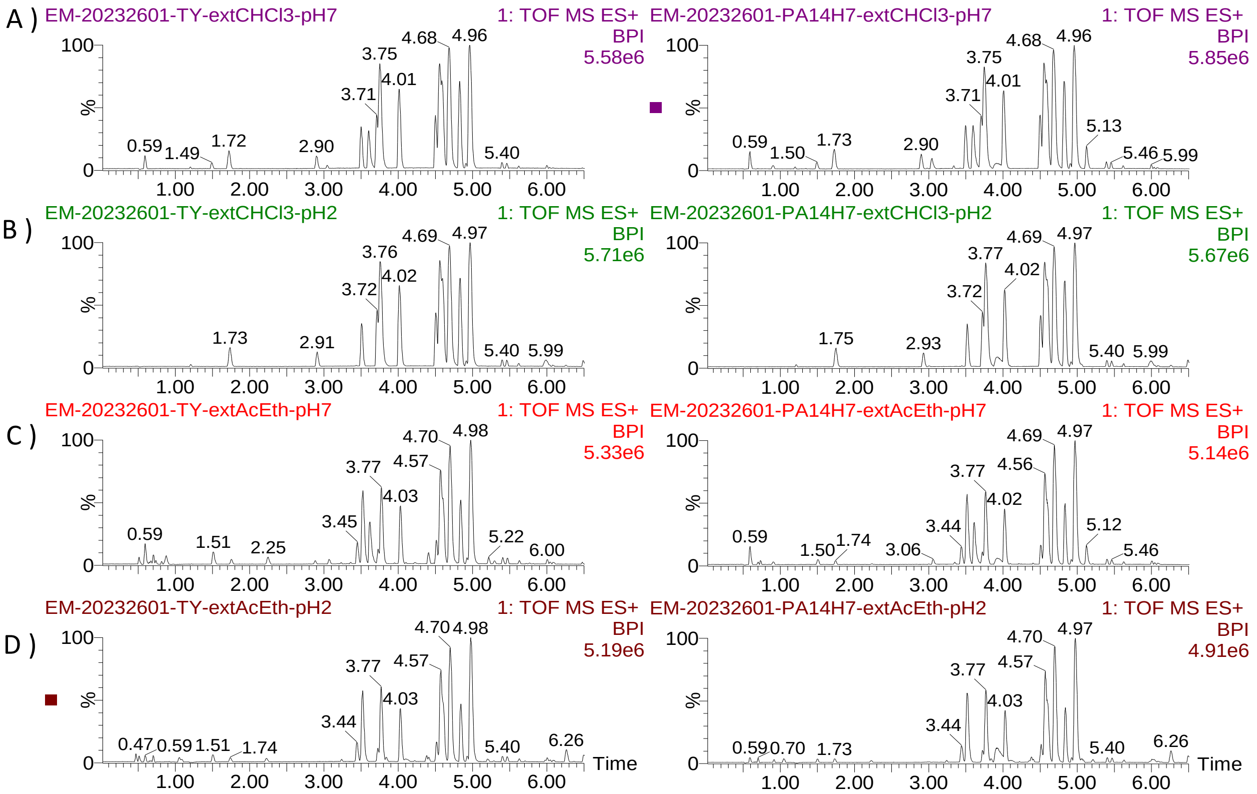

Figure S4: LC-MS (ESI+) comparison of TY culture medium (left) and CFS-PA14H7 (right) BPI chromatograms of organic phases after extraction with chloroform at pH 7 (A), chloroform at pH 2 (B), ethyl acetate pH 7 (C) or ethyl acetate pH 2 (D). Extraction performed on the same batch of TY and PA14H7 filtered supernatant.

Table S2: Optical Density at 600 nm (OD 600nm) measurement of organic and aqueous phases of CFS-PA14H7 extract with chloroform or ethyl acetate at pH 2 or pH 7 after 0 h, 24 h and 48 h of incubation with *D. solani*.

			Organic phase			Aqueous phase		
**Extraction method of CFS-PA14H7**		**dilution**	**t0**	**t24**	**t48**	**t0**	**t24**	**t48**
**Chloroform**	**pH 2**	control	0.043	0.044	0.044	0.040	0.040	0.040
		pur	0.058	0.060	0.071	0.043	0.042	0.043
		1/2	0.052	0.055	0.055	0.041	0.041	0.041
		1/4	0.044	0.044	0.045	0.041	0.040	0.040
		1/10	0.045	0.044	0.045	0.040	0.041	0.041
		1/20	0.042	0.042	0.042	0.041	0.042	0.041
		1/100	0.042	0.131	0.189	0.041	0.174	0.198
		1/200	0.041	0.144	0.213	0.040	0.176	0.178
		1/1000	0.042	0.131	0.211	0.041	0.120	0.201
	**pH 7**	control	0.044	0.044	0.044	0.052	0.041	0.041
		pur	0.070	0.066	0.061	0.047	0.213	0.277
		1/2	0.053	0.050	0.049	0.042	0.170	0.222
		1/4	0.046	0.045	0.045	0.040	0.147	0.218
		1/10	0.045	0.043	0.043	0.042	0.147	0.209
		1/20	0.043	0.042	0.044	0.041	0.171	0.185
		1/100	0.041	0.137	0.178	0.041	0.185	0.180
		1/200	0.041	0.188	0.193	0.040	0.189	0.182
		1/1000	0.041	0.126	0.206	0.041	0.133	0.198
**Ethyl acetate**	**pH 2**	control	0.041	0.042	0.041	0.040	0.041	0.041
		pur	0.053	0.053	0.060	0.043	0.287	0.269
		1/2	0.046	0.047	0.047	0.042	0.163	0.245
		1/4	0.046	0.046	0.046	0.041	0.154	0.238
		1/10	0.043	0.043	0.043	0.042	0.153	0.231
		1/20	0.043	0.043	0.044	0.044	0.162	0.222
		1/100	0.042	0.094	0.166	0.041	0.157	0.214
		1/200	0.041	0.146	0.203	0.041	0.157	0.198
		1/1000	0.042	0.161	0.202	0.042	0.164	0.206
	**pH 7**	control	0.043	0.043	0.045	0.039	0.040	0.040
		pur	0.045	0.045	0.045	0.042	0.167	0.386
		1/2	0.042	0.043	0.043	0.040	0.139	0.282
		1/4	0.041	0.041	0.041	0.039	0.141	0.226
		1/10	0.042	0.041	0.041	0.039	0.129	0.209
		1/20	0.041	0.042	0.042	0.039	0.128	0.208
		1/100	0.040	0.119	0.182	0.041	0.156	0.215
		1/200	0.040	0.129	0.213	0.039	0.172	0.215
		1/1000	0.040	0.133	0.202	0.041	0.149	0.198

Legend:
- no growth in lliquid medium
- growth in liquid medium

Negative controls are used to verify the absence of contamination of extracts and medium used. The combinination where no bacterial growth is observed after 48 of incubation (green) are enumerated on Petri dish for the determination of bactericidal/bacteriostatic effect.

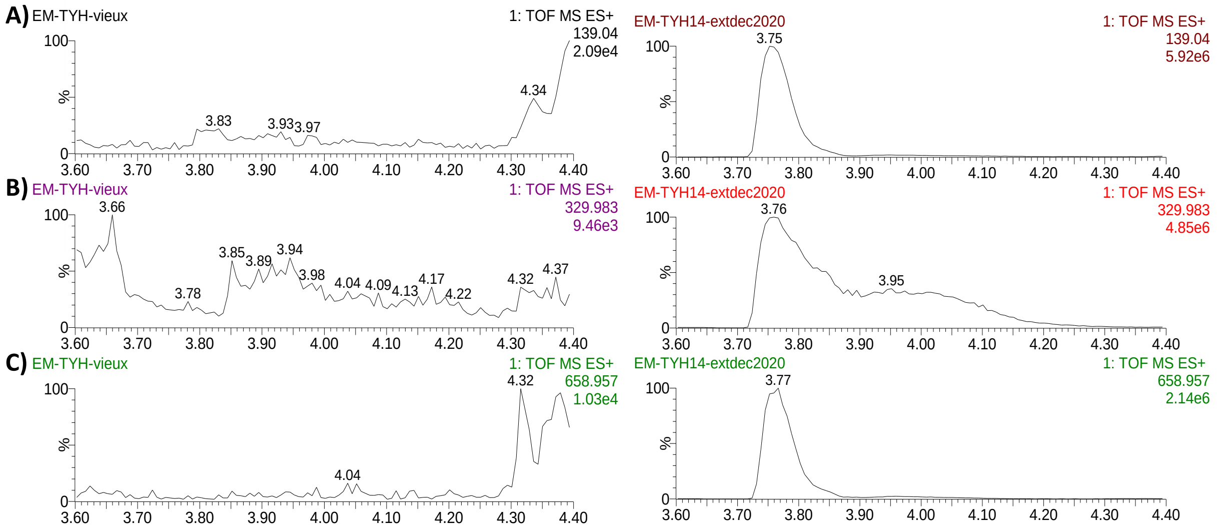

Figure S5: The reconstituted ion current (RIC) of the ions observed between 3.6 and 4.6 min at m/z 139.040 (A), m/z 329.983 (B) and m/z 658.957 (C) in TY culture medium (left) and CFS-PA14H7 (right).

Table S3: Summary of markers present in CFS-PA14H7 and absent in TY medium analyzed with marker lynx (Waters, Masslynx V4.2).

Ret. Time	m/z	Included	Saturated	EM-081221-MeOH-a	EM-081221-extTY-	EM-081221-extA14H7-CHCl3-a	EM-081221-MeOH-b	EM-081221-extTY-	EM-081221-extA14H7-CHCl3-b	EM-081221-MeOH-c	EM-081221-extTY-	EM-081221-extA14H7-CHCl3-c	MOY-MeOH	MOY-extTY	MOY-ext CFS-PA14H7
**3.5814**	**329.9821**	Yes	No	0	0	64.2733	0	0	74.0706	0	0	67.0571	**0.0000**	**0.0000**	**68.4670**
**3.5631**	**139.0393**	Yes	No	0	0	26.5547	0	0	28.0006	0	0	29.36	**0.0000**	**0.0000**	**27.9718**
**2.6099**	**107.0497**	Yes	No	0	0	0	0	0	25.5209	0	0	26.8061	**0.0000**	**0.0000**	**17.4423**
**5.4185**	**473.2756**	Yes	No	0	0	15.6818	0	0	16.2169	0	0	16.4323	**0.0000**	**0.0000**	**16.1103**
**3.6385**	**197.1290**	Yes	No	0	0	0	0	0	26.6137	0	0	21.5049	**0.0000**	**0.0000**	**16.0395**
**4.8521**	**430.7162**	Yes	No	0	0	12.8648	0	0	13.6696	0	0	13.8827	**0.0000**	**0.0000**	**13.4724**
**4.6698**	**860.4260**	Yes	No	0	0	14.2627	0	0	9.6497	0	0	14.3276	**0.0000**	**0.0000**	**12.7467**
**4.8537**	**609.3388**	Yes	No	0	0	12.3265	0	0	12.219	0	0	12.0869	**0.0000**	**0.0000**	**12.2108**
**4.2119**	**540.3383**	Yes	No	0	0	11.8303	0	0	11.9083	0	0	11.9421	**0.0000**	**0.0000**	**11.8936**
**4.8505**	**860.4267**	Yes	No	0	0	10.9442	0	0	11.7755	0	0	12.3206	**0.0000**	**0.0000**	**11.6801**
**4.9946**	**746.3832**	Yes	No	0	0	10.7787	0	0	11.7357	0	0	11.8424	**0.0000**	**0.0000**	**11.4523**
**5.2181**	**657.3956**	Yes	No	0	0	10.4574	0	0	10.4247	0	0	10.4402	**0.0000**	**0.0000**	**10.4408**

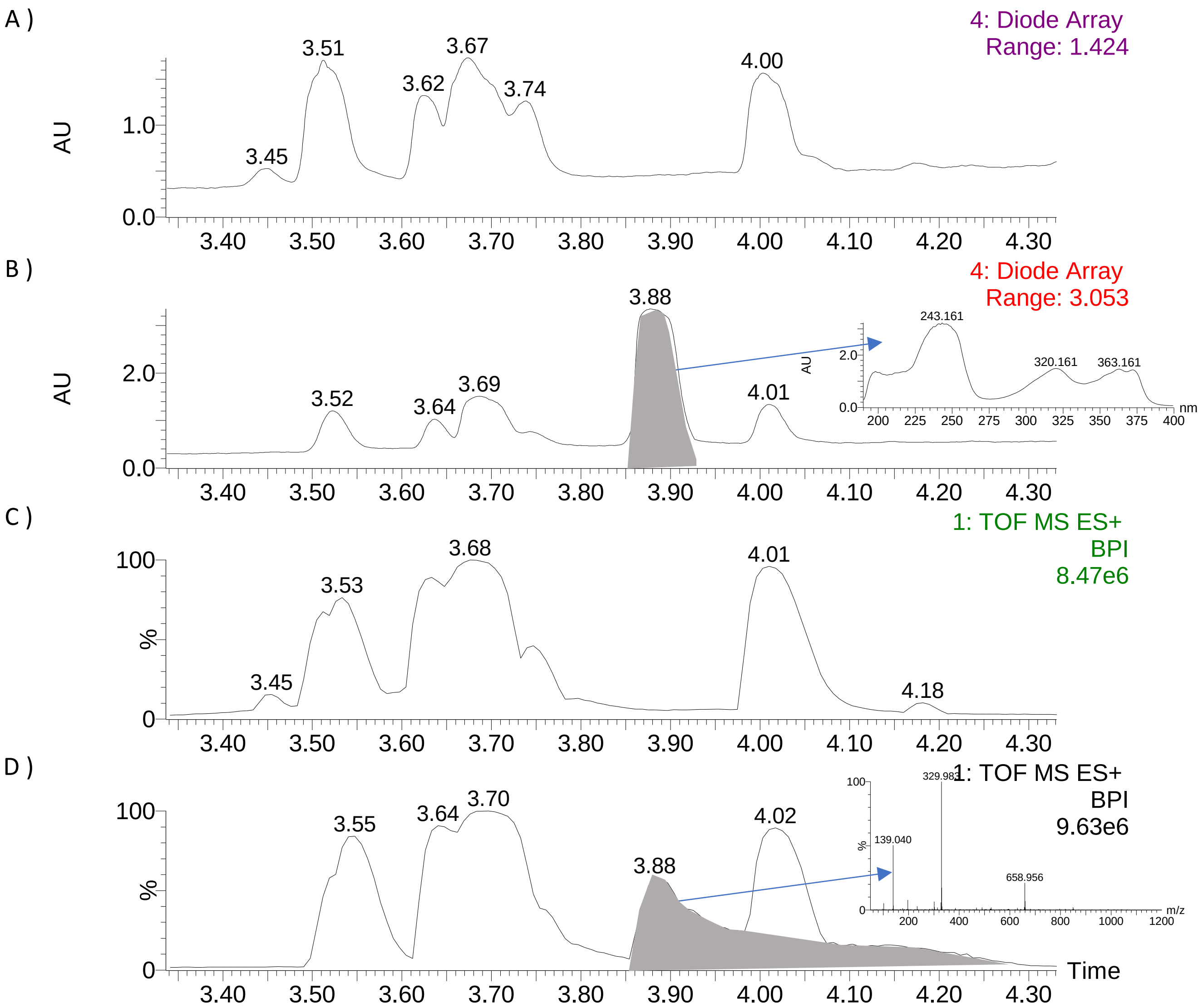

Figure S6: UV trace corresponding to the maximum of absorbance of the iron complex (B), absent in TY UV trace (A). Corresponding LC-MS (ESI+) BPI chromatograms for TY culture medium (C) and CFS-PA14H7 (D) highlighting the presence of the complex at m/z 329.983.

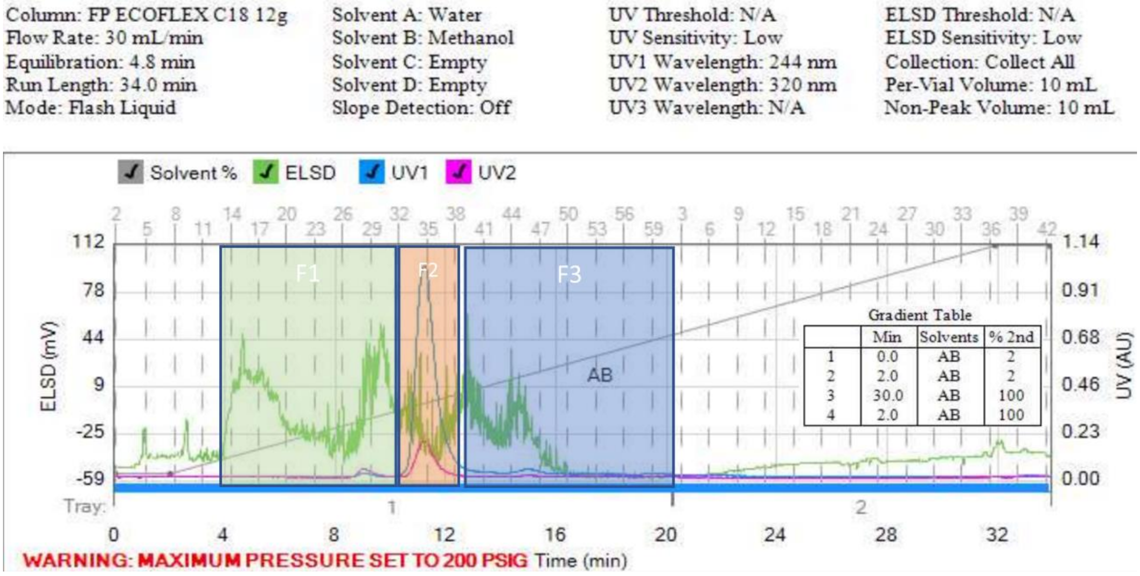

Figure S7: Ultra-Violet (UV) at 224 nm (blue) and 320 nm (purple) and Evaporative Light Scattering Diffusion (ELSD) (green) chromatograms of the flash chromatography purification of CFS-PA14H7 using a C18 column. Fraction F1 is highlighted in green, F2 in orange, and F3 in blue.

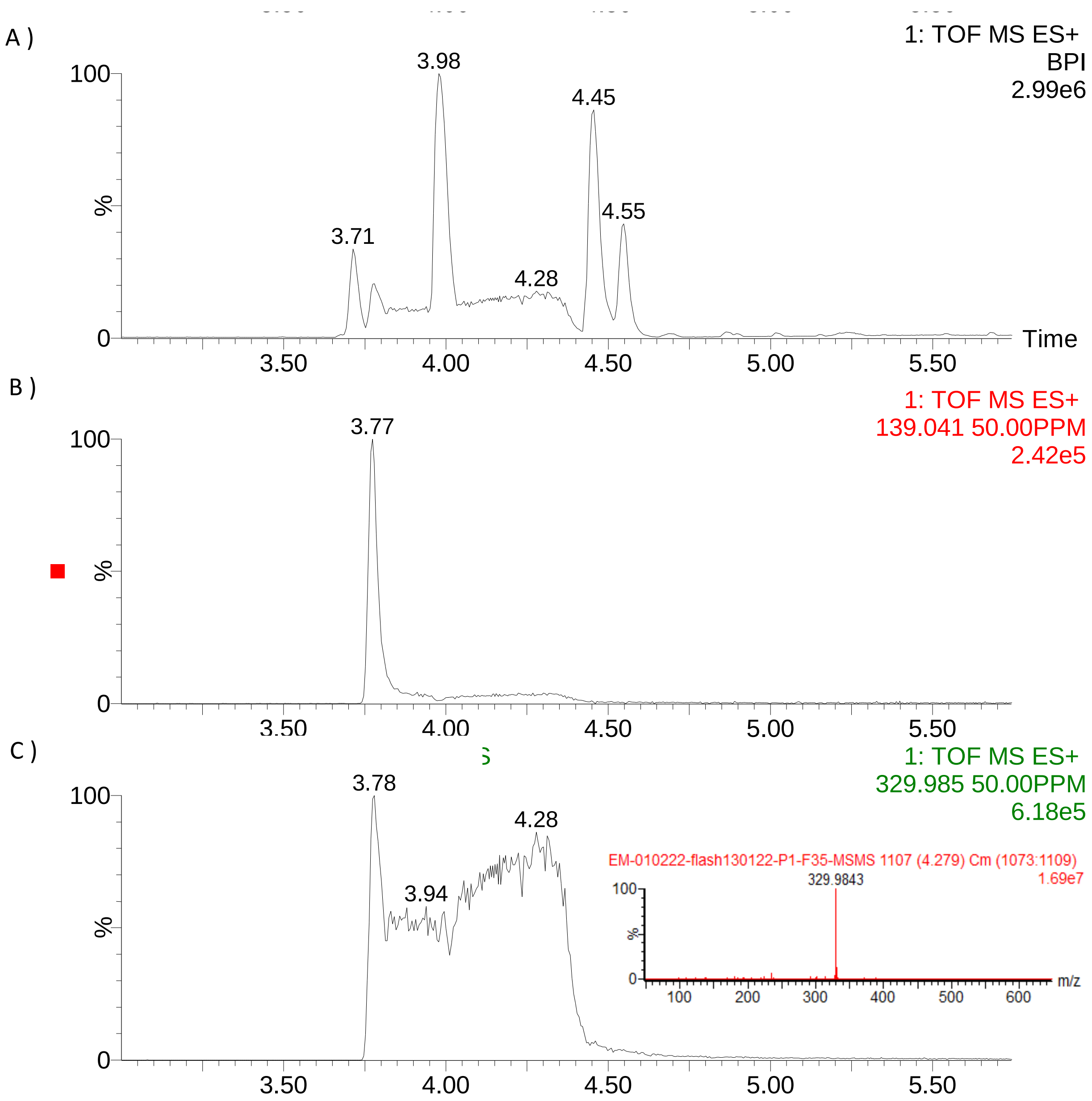

Figure S8: LC-MS (ESI+) chromatograms of fraction F2 resulting from CFS-PA14H7 purification by flash chromatography, BPI trace (A), RIC of the ions at m/z 139.040 (B) and m/z 329.983 (C). The purified iron complex is effectively detected between 3.80 and 4.40 min. The corresponding mass spectra are also depicted.

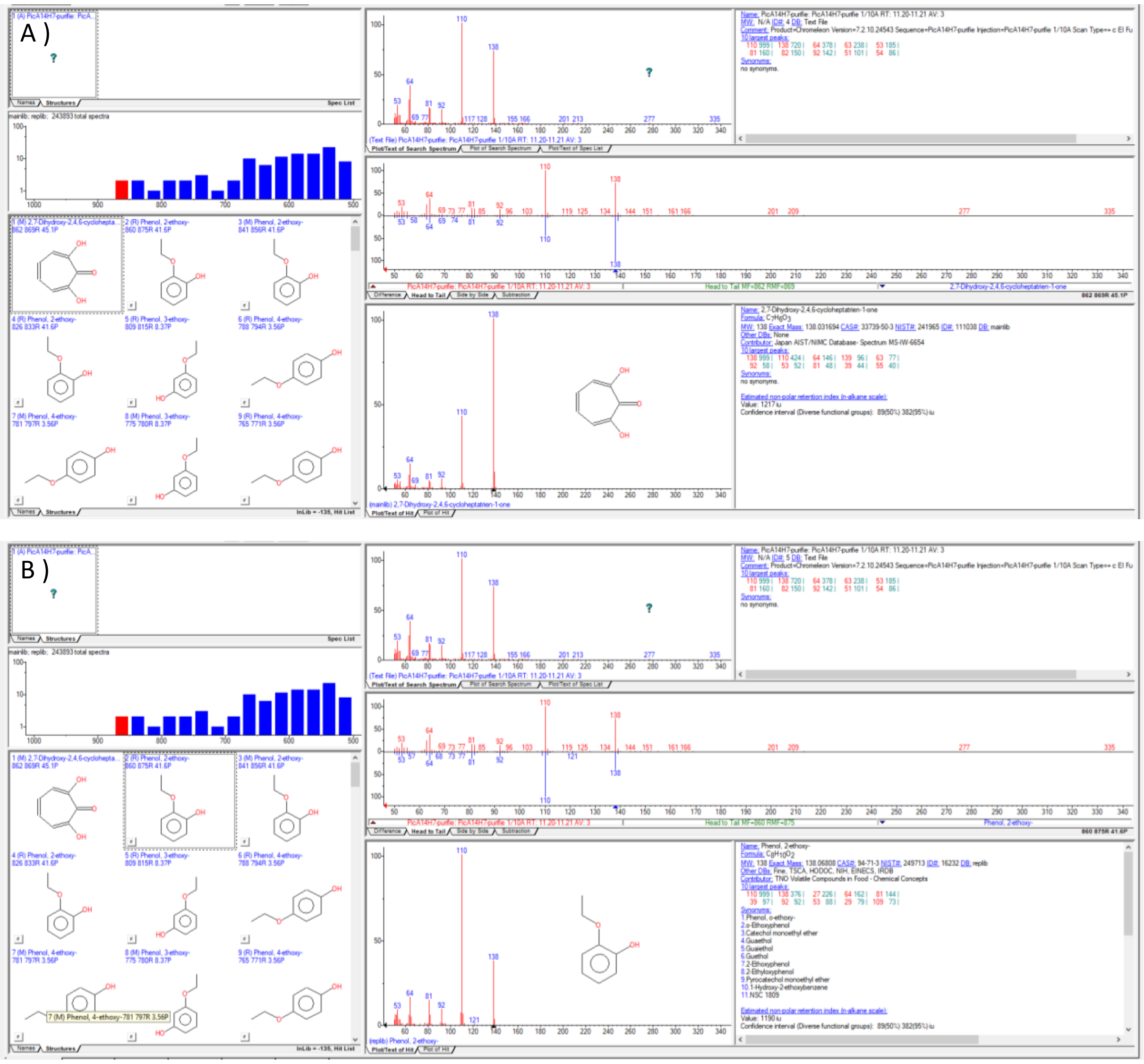

Figure S9: EI NIST library spectra of the different hypothesis for the molecular ion observed at m/z 138. 7-HT (A) and 2-ethoxyphenol and isomers (B).

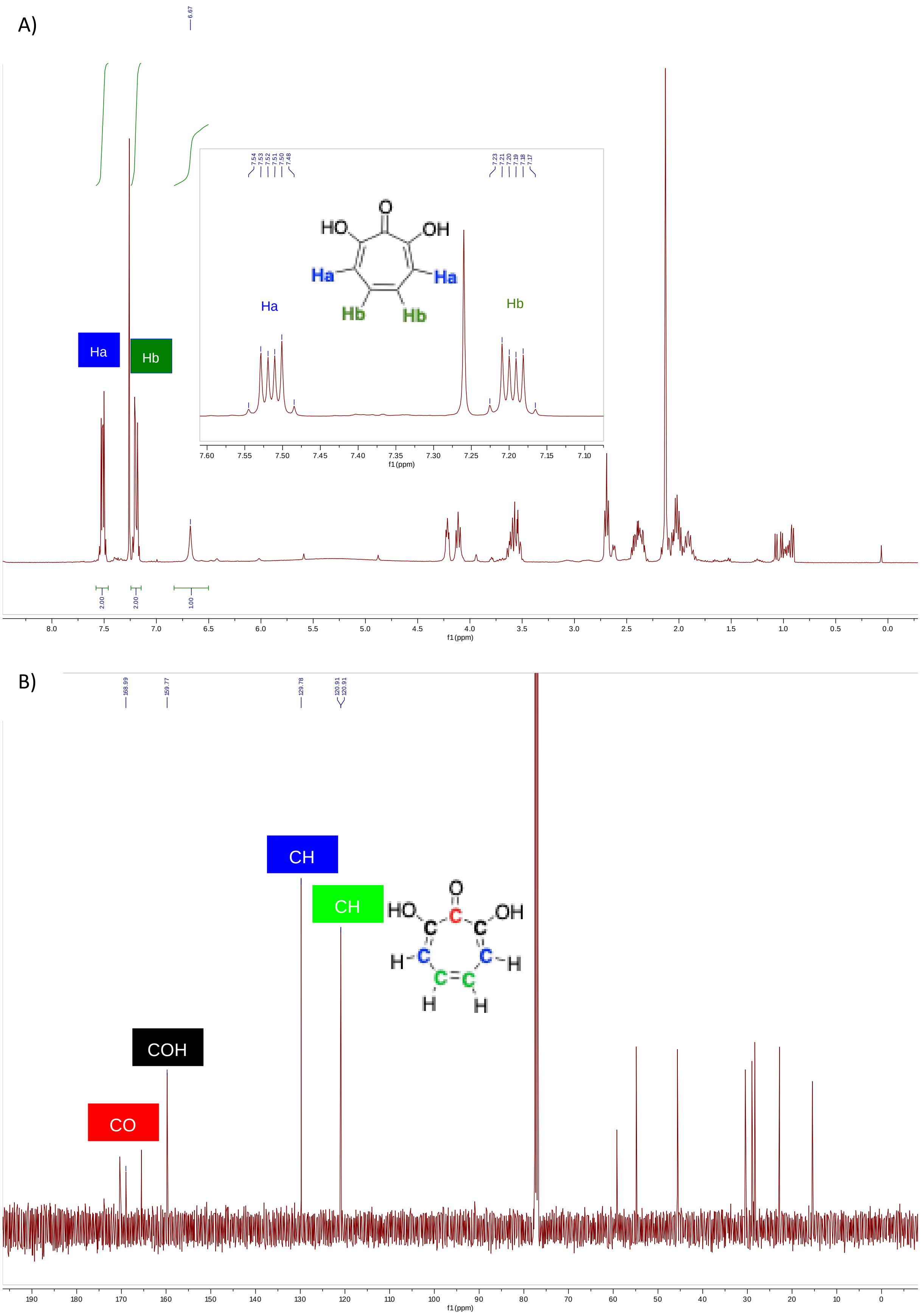

Figure S10: Detailed NMR spectra of fraction F2 resulting from CFS-PA14H7 purification by flash chromatography: (A) ^{1}H NMR (400 MHz) and (B) ^{13}C NMR (100 MHz).

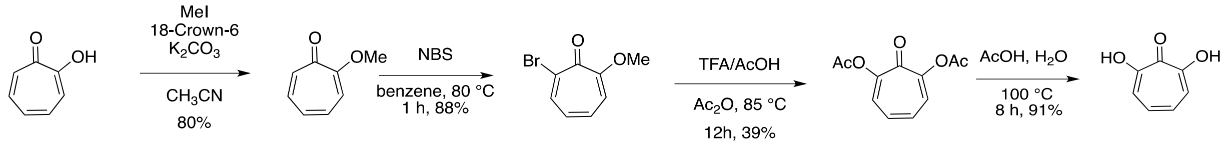

Figure S11: Synthesis of 7-hydroxytropolone starting from commercial tropolone. From Winter, N.; Trauner ; D. *J. Am. Chem. Soc.* **2017**, *139*, 11706-11709 and Takeshita, H.; Mori, A. *Synthesis* **1985**, 578-579. The final product was recrystallized in toluene.

A)

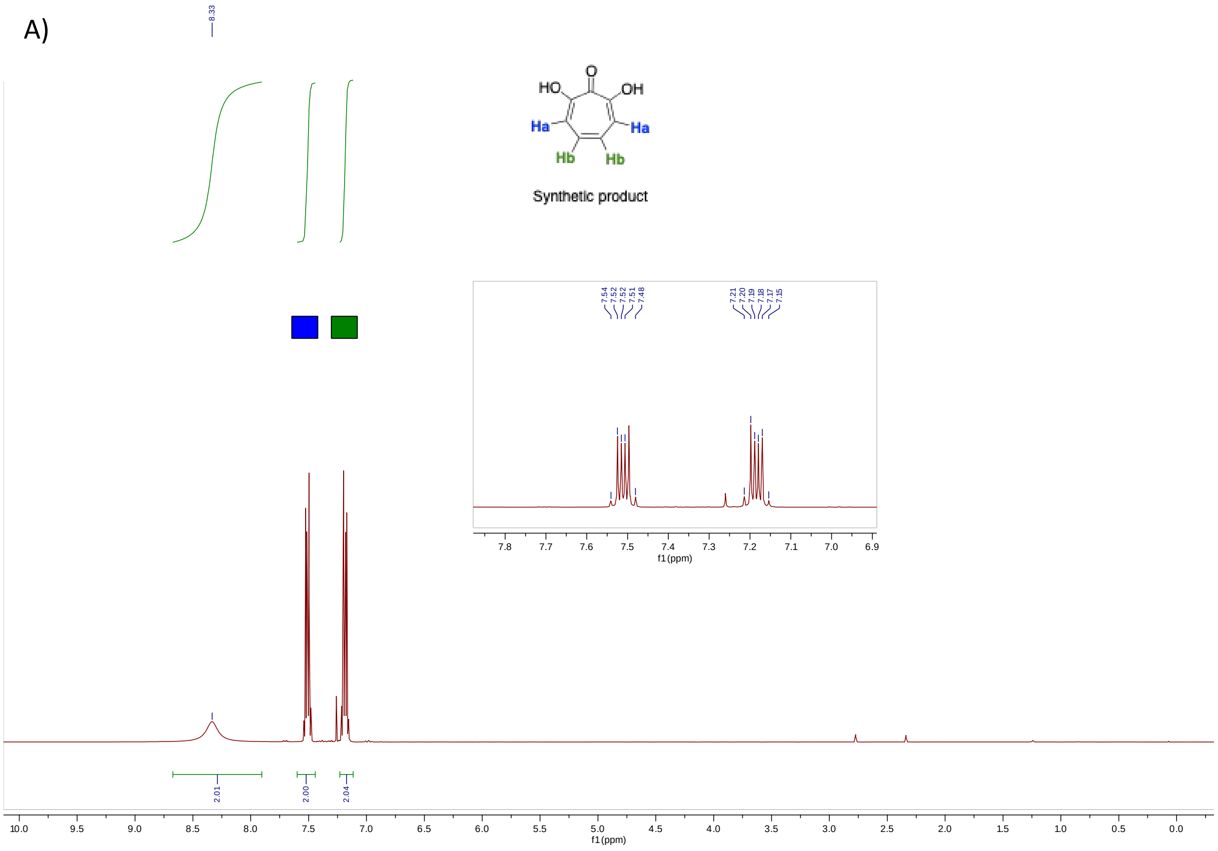

B)

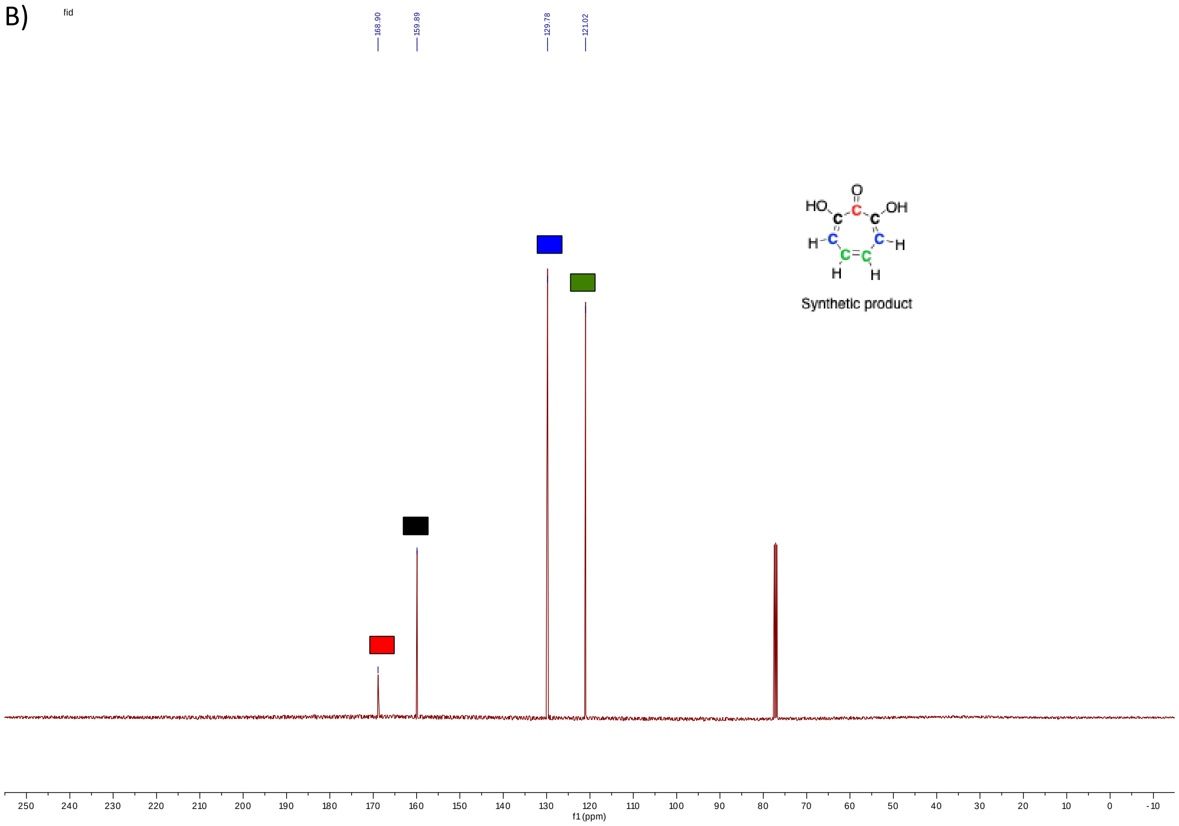
fid
168.90
159.89
129.78
121.02
HO
OH
O
H
Synthetic product
f1 (ppm)

C)

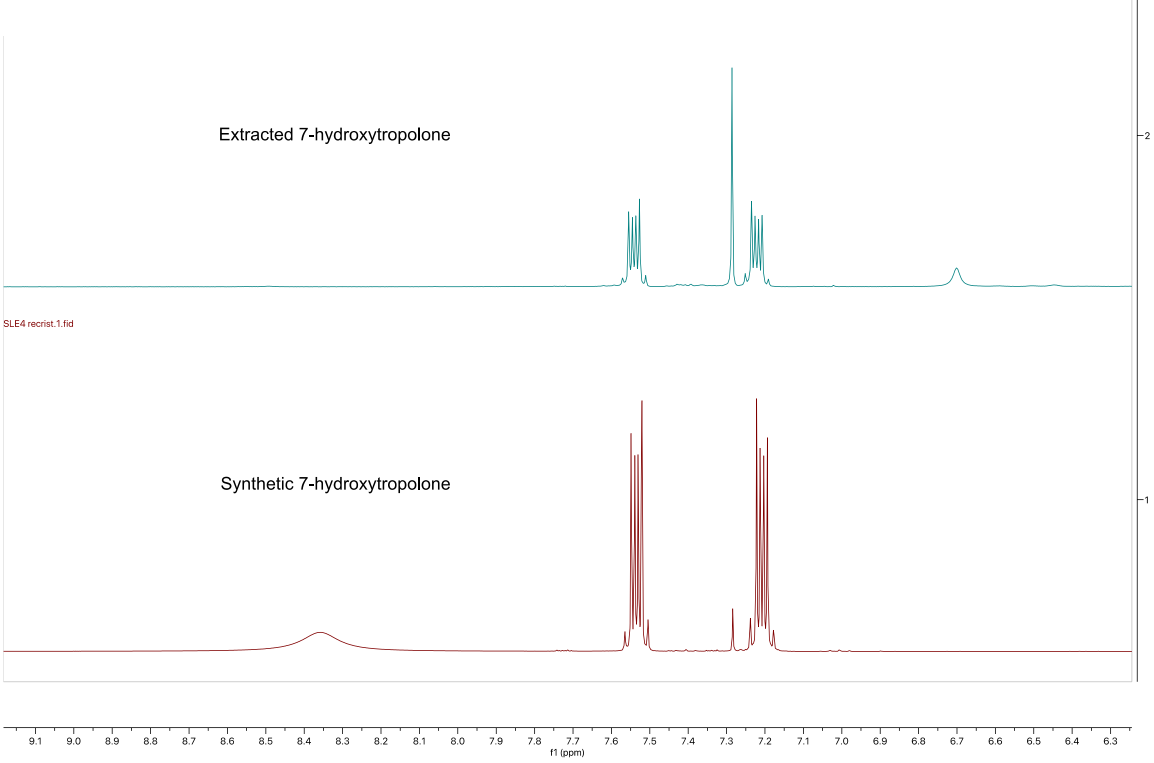
Extracted 7-hydroxytropolone
SLE4 recrist.1.fid
Synthetic 7-hydroxytropolone
f1 (ppm)

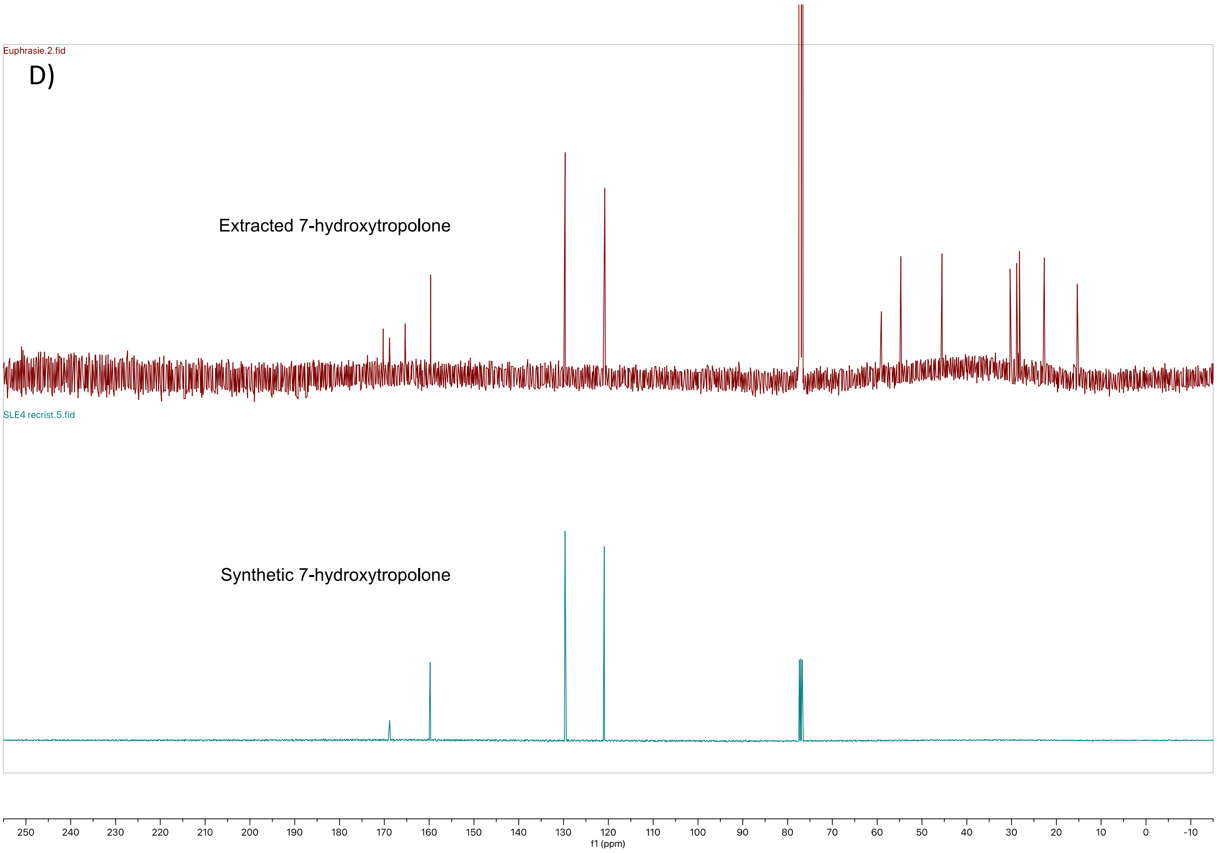

Figure S12: (A) ^{1}H NMR and (B) ^{13}C NMR spectra of 7-HT obtained by synthesis and (C) ^{1}H NMR spectra and (D) ^{13}C NMR spectra comparison of fraction F2 resulting from CFS-PA14H7 purification by flash chromatography (top) and synthetic 7-HT (bottom) between 6.3 and 9.1 ppm.

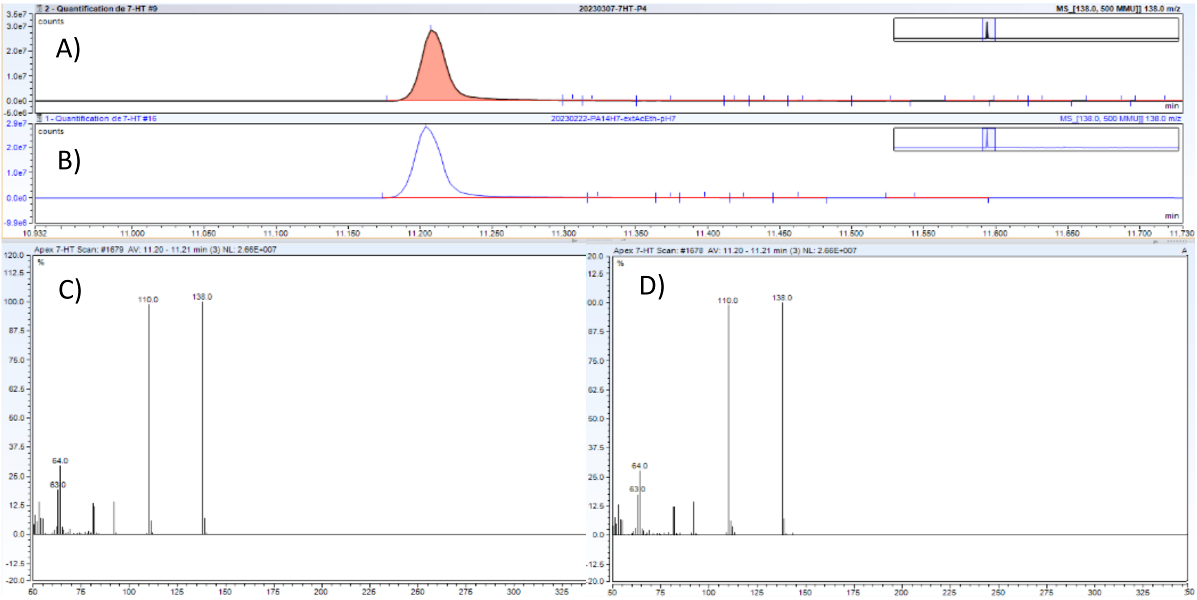

Figure S13: GC-MS chromatograms and spectra of 7-HT obtained by synthesis (A, C) and of fraction F2 resulting from CFS-PA14H7 purification by flash chromatography (B, D).

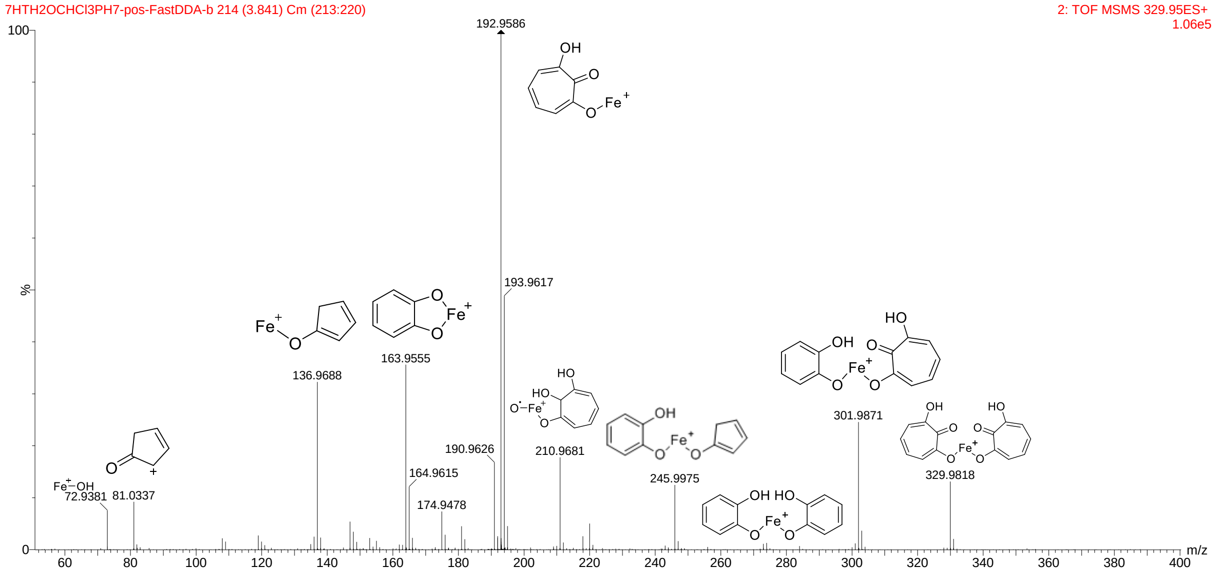

Figure S14: Proposal fragmentation pattern of 7-HT iron complex ion (m/z 329.983) by MS/MS (30 eV).

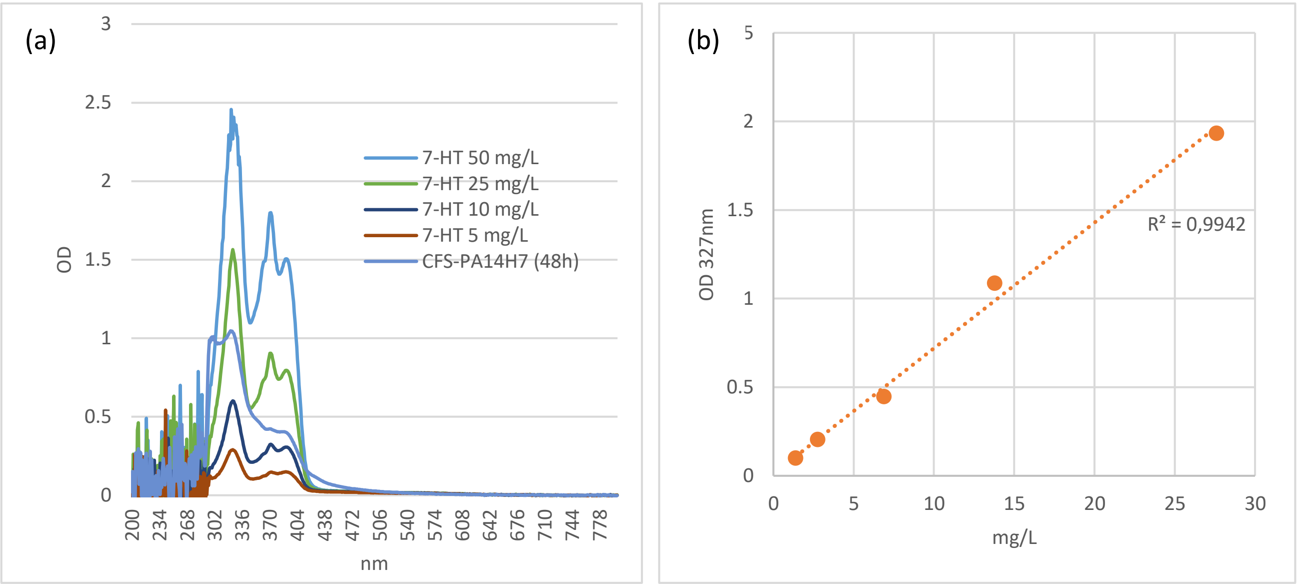

Figure S15: (a) UV spectrum of CFS-PA14H7 (1L, 48h) and of 7-HT of various concentrations in solution in TY (b) calibration curve of 7-HT in TY solution, at 327 nm ranging from 1.4 to 27.6 mg/L.

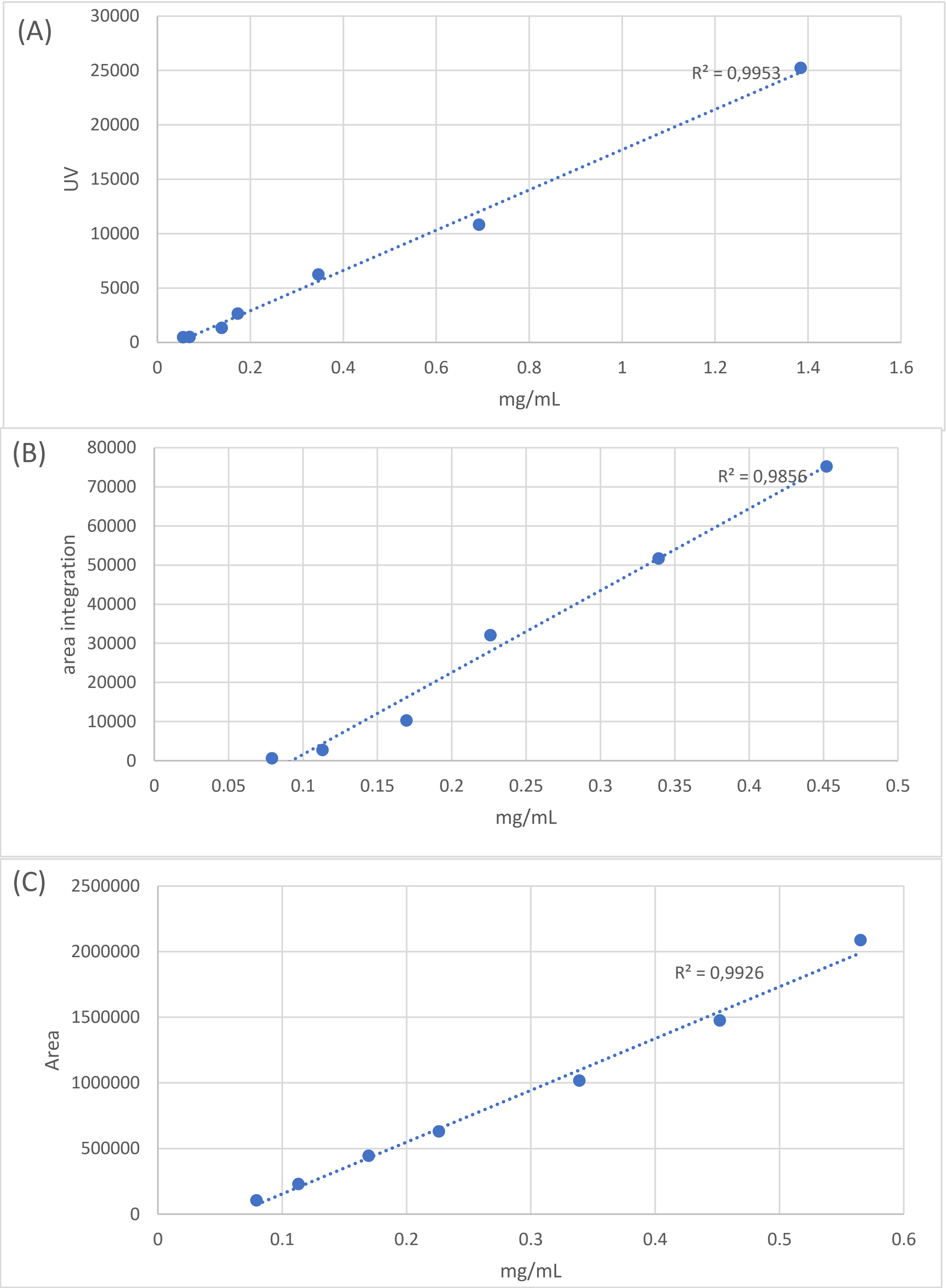

Figure S16: Standard curve performed with 7-HT synthetic molecule range from 0.07 mg/mL and 0.56 mg/mL according to LC-UV (320nm) (A), LC-MS (m/z 329.983) (B) and GC-MS (m/z 138) analysis (C).
